# Development and validation of the dysarthria impact scale: a patient-reported outcome for motor speech disorders

**DOI:** 10.1007/s00415-026-13740-1

**Published:** 2026-03-10

**Authors:** Adam P. Vogel, Lisa Graf, Merit Weiß, Cheuk S. J. Chan, Graham Hepworth, Matthis Synofzik

**Affiliations:** 1 Department of Neurodegenerative Diseases, Hertie-Institute for Clinical Brain Research and Center of Neurology, University of Tübingen, Tübingen, Germany; 2 Speech Pathology, School of Health Sciences, The University of Melbourne, Melbourne, Australia; 3 Redenlab Ltd., Melbourne, Australia; 4 Statistical Consulting Centre, The University of Melbourne, Melbourne, Australia; 5 German Center of Neurodegenerative Diseases (DZNE), Tübingen, Germany; 6 550 Swanston Street, Parkville, VIC 3010 Australia

**Keywords:** Speech, Dysarthria, Patient-reported outcome, Clinical trials, Questionnaire, Survey

## Abstract

**Background:**

Impaired speech due to dysarthria significantly impacts quality of life. Patient-reported outcomes (PROs) offer critical insight into the lived experience of communication disability and are central to regulatory frameworks for patient-focused drug development.

**Objectives:**

To develop and validate the Dysarthria Impact Scale (DIS), a brief PRO designed to assess the impact of motor speech disorders on quality of life across neurological conditions.

**Methods:**

A multi-site, cross-sectional study was conducted with 244 participants, including individuals with Huntington’s disease, Parkinson’s disease, hereditary ataxias, and head and neck cancer, and healthy controls. The 22-item DIS was developed using expert input and patient feedback and evaluated alongside reference tools (Voice Handicap Index and SF-36). Item reduction procedures yielded two shorter versions (DIS-17 and DIS-6). Validity, reliability, and sensitivity/specificity analyses were performed, and minimal clinically important differences (MCIDs) were estimated using distribution-based methods.

**Results:**

All DIS versions showed strong convergent validity with the VHI (*r* = −0.85) and SF-36 (*r* = 0.72) and were correlated with blinded perceptual speech ratings. DIS-17 and DIS-6 achieved comparable sensitivity (0.93 and 0.88) and specificity (0.84 and 0.86, respectively). Test–retest reliability was high (*r* = 0.98), with estimated MCIDs and within-subject variability provided. Group differences were observed, with lower DIS scores in ataxia and Parkinson’s disease compared to Huntington’s disease.

**Conclusions:**

The DIS is a valid, reliable, and practical PRO for quantifying the impact of dysarthria on quality of life. Longitudinal responsiveness remains to be established.

**Electronic supplementary material:**

The online version of this article (10.1007/s00415-026-13740-1) contains supplementary material, which is available to authorized users.

## Introduction

Brain disease or injury causing motor dysfunction frequently results in disordered speech. This impairment can lead to daily disadvantage and stigmatization [1–3]. Dysarthria is the most common neurological speech disorder. It is characterized by sensorimotor impairment in one or multiple speech subsystems including respiration, phonation, articulation, resonance, and prosody. Deficits in any or all these subsystems can reduce intelligibility (ability to be understood) and naturalness (deviation from a healthy norm) and impact overall communicability. It is established that communication deficits trigger altered self-identity [4], impede social [5] and professional interactions [3], and lead to social marginalization [6]. Seventy percent of people with a communication disorder are unemployed or in the lowest income brackets [7]. These conditions have profound health, psychosocial, and economic consequences, yet tools to evaluate the impact of dysarthria on patients are limited.

The nature and impact of dysarthria on the speaker vary based on factors including site, size, and type of lesion or neurological damage. For example, neurodegenerative diseases such as Huntington’s disease, Parkinson’s disease, or hereditary ataxia lead to a progressive decline in articulation accuracy, vowel distortion, altered resonance, and slower or altered rate of speech [8–10]. Stroke or traumatic brain injury, neurodevelopmental conditions (e.g., FOXP2), or structural abnormalities resulting from head and neck conditions (e.g., pharyngeal cancer) may present with static deficits across subsystems [11–14]. Both neurodegenerative and non-progressive etiologies appear to be amenable to treatment [15–22]; however, there are no pharmaceutical therapies specifically designed to improve speech.

Tools specifically built for measuring dysarthria-related quality of life available to clinicians and researchers include the Dysarthria Impact Profile [23], the QOL-DyS [24], and the Living with dysarthria: evaluation of a self-report questionnaire [25]. These tools differ in scope, validation protocols, cohort composition, question design, length, and language availability. While each has strengths, none simultaneously meets the requirements of brevity, cross-diagnostic applicability, multilingual use, and validation against external reference measures. For example, some tools are validated across heterogeneous clinical cohorts (e.g., Living with dysarthria), some are relatively lengthy and too complex for routine clinical or trial use (e.g., Dysarthria Impact Profile, which is too long for many protocols), some were not validated against standardized comparators (e.g., [24]), and some employ both negative and positive questioning (e.g., Dysarthria Impact Profile), which is confusing for individuals both with and without a cognitive impairment.

A comprehensive assessment of motor speech impairment requires the use of patient-reported outcomes (PROs) specifically focused on dysarthria-related quality of life. The term PRO is used here to emphasize the construct being measured, while the DIS itself functions as a patient-reported outcome measure (PROM). Also, patient-focused drug development requires trial outcomes (e.g. of speech) to be anchored to outcomes shown meaningful to and by patients and their network. Presented in another way, the US Food and Drug Administration (FDA) describes clinical outcome assessments (COAs) as measures that directly quantify what matters most to individuals, including how they function and feel [26, 27]. Consequently, assessment of any impact of treatment, either pharmaceutical or behavioral, or disease (either neurodegenerative or non-progressive) should include a combination of objective and subjective tests [28]. In clinical trials, tests should be brief, repeatable, and suitably motivating to ensure completion at regular intervals [29, 30]. In this study, speech-related quality of life is conceptualized as the perceived impact of dysarthria on daily functioning, communicative participation, and psychosocial well-being, rather than global health-related quality of life or diagnostic severity. Here we describe development and validation of the Dysarthria Impact Scale, as a novel brief PRO for assessing the impact of dysarthria for people with motor speech impairment, to be used in clinical assessments, natural history studies, or treatment trials.

## Methods

The protocol was approved by the Institutional Review Boards of The University of Melbourne Human Ethics Committee (2024-11758-51349-6) and University of Tübingen (003/2015BO2) in accordance with the Declaration of Helsinki.

### Tool development

Rather than undertaking de novo qualitative item generation, we adopted a theory and literature-driven approach to derive items reflecting key domains of dysarthria impact, supplemented by targeted patient and clinician review to ensure relevance, clarity, and face validity. The COSMIN (COnsensus-based Standards for the selection of health status Measurement INstruments) risk of bias checklist was used to optimize methodological quality and limit risk of bias of the proposed tool [31, 32]. The a priori clinometric properties targeted in the tool design focused on integrating adequate reliability and validity, minimizing measurement error and maximizing responsiveness to symptom severity. It was considered important that the proposed tool was brief, easy to complete, and suitable for use in a variety of clinical settings and languages.

We first conducted a review of the literature to identify comparable patient-reported outcomes in communication research and clinical practice. This included examination of PROMs designed for use in aphasia (language impairment), motor speech (including dysarthria and apraxia), and voice, and general communication impairment. This identified several tools with overlapping features and themes relevant to our own tool, including the Communicative Participation Item Bank [33], the Voice Handicap Index, [34] and the Dysarthria Impact Profile [23] (See Supplementary Materials S1 for detail). However, no existing tools met all requirements for use. A small purposive sample of individuals with progressive dysarthria (Friedreich ataxia; *n* = 5) was consulted to review item relevance, wording, and comprehensibility, rather than to undertake primary qualitative item generation (See Supplementary Materials S2). Patient involvement at this stage was limited to feasibility review rather than formal item rating, reflecting pragmatic constraints of a multi-site study. Combining information from existing tools and people living with progressive dysarthria, we focused on developing questions across three established domains of communication: physical functioning, emotional functioning, and functional impact of dysarthria.

The first series of 29 items was created across these three domains. Clinical expert review involved two speech–language pathologists with experience in motor speech disorders, a neurologist, and a general physician. Items were independently rated for relevance, clarity, and redundancy. Iterative consensus discussions were used to remove or refine items judged to be overlapping, ambiguous, or insufficiently aligned with the intended construct, resulting in reduction from 25 to 22 items. A trial version was condensed to 22 items following a final review by content experts and a trial with the clinical cohorts (see Supplementary Materials S4 for trial items). Subsequent shorter versions of the tool were also tested, namely DIS 17-item [DIS-17] and DIS 6-item [DIS-6]. Item wording was adapted from existing instruments and refined to ensure consistent directionality, simple syntax, and relevance to dysarthria-related daily communication, rather than voice- or language-specific impairment.

### Participants

244 participants were recruited to the study (52.9% female). Cohorts included individuals with Huntington’s disease (*n* = 45), Friedreich ataxia (*n* = 38), Parkinson’s disease (*n* = 31), spinocerebellar ataxia (*n* = 21), or head and neck cancer (*n* = 20) [participants were recruited from the head and neck ward and were included if they presented with any speech impairment resulting from their condition]. The head and neck cancer cohort was included as a non-neurodegenerative comparator group to examine the cross-etiology validity of the DIS in dysarthria arising from structural rather than neurological causes. Other diagnoses included cerebellar ataxia, neuropathy, vestibular areflexia syndrome (CANVAS) or multiple system atrophy-cerebellar (MSA-C) (*n* = 19). Seventy age- and sex-matched controls also participated. See Table 1 for demographic and clinical information. Recruitment and testing were undertaken in Melbourne, Australia, and Tübingen, Germany. Clinical participants were excluded from the degenerative disease cohorts if they had comorbid neurological diseases known to impact communication (e.g., stroke, multiple sclerosis); clinical symptoms other than those caused by their primary disease; lacked competency in English or German (depending on site); a history of alcohol or drug abuse that required medical intervention; or a history of learning disability and/or intellectual impairment. Eligibility for inclusion as a healthy control required having no family history of a neurological disease and had unremarkable cognition, speech, and language function, as assessed by a speech pathologist. Cognition was assessed using the Montreal Cognitive Assessment (MoCA) [35]. All subjects provided informed consent to participate in the study.

**Table 1:** Clinical and demographic information for the cohort

	All (*N* = 244,♀ = 129)	FRDA (*N* = 38,♀ = 22)	SCA (*N* = 21) ♀ = 10)	Other ataxias (*N* = 19,♀ = 14)	HD (*N* = 45,♀ = 24)	PD (*N* = 31,♀ = 9)	H&N (*N* = 20,♀ = 5)	HC (*N* = 70,♀ = 45)	Kruskal–Wallis (*p*)
Age at assessment yrs ± SD (range)	54.4 ± 15.6 (20–88)	44.8 ± 11.4 (20–79)	58.5 ± 9.9 (41–72)	59.4 ± 12.0 (31–79)	46.4 ± 14.6 (22–68)	68.6 ± 6.2 (57–79)	65.8 ± 7.6 (53–88)	52.6 ± 2.8 (20–88)	< 0.001
Age at onset yrs ± SD (range)	53.1 ± 14.2 (18–87)	43.9 ± 15.4 (18–64)	33.2 ± 15.9 (18–59)	48.7 ± 16.2 (25–65)	49.6 ± 13.8 (25–67)	54.7 ± 14.2 (18–87)	64.0 ± 8.1 (52–87)	NA	< 0.001
Disease duration ± SD (range)	8.9 ± 8.0 (0–33)	10.3 ± 4.7 (3–15)	16.4 ± 9.8 (18–59)	9.1 ± 9.7 (1–29)	5.0 ± 3.6 (1–15)	13.9 ± 7.3 (2–33)	1.8 ± 1.7 (0–5)	NA	< 0.001
Speech severity score (intelligibility)	1.2 ± 1.2 (0–4)	2.3 ± 0.9 (1–4)	1.6 ± 1.1 (0–4)	1.9 ± 0.9 (0–4)	0.9 ± 1.0 (0–4)	1.7 ± 1.2 (0–4)	1.8 ± 1.3 (0–4)	0 ± 0 (0–0)	< 0.001 ^b^
Speech severity score (naturalness)	1.3 ± 1.2 (0–4)	2.3 ± 1.1 (0–4)	1.9 ± 1.1 (0–4)	2.0 ± 1.1 (0–3)	1.3 ± 0.9 (0–3)	1.9 ± 1.0 (0–4)	1.3 ± 1.0 (0–4)	0 ± 0 (0–0)	< 0.001 ^b^
MoCA total score ± SD (range)	25.8 ± 3.6 (11–30)	25.6 ± 1.6 (23–27)	24.2 ± 2.4 (21–27)	23.7 ± 3.0 (19–29)	25.4 ± 5.2 (11–30)	25.5 ± 2.8 (19–30)	23.8 ± 2.0 (21–28)	27.4 ± 2.8 (22^c^–30)	< 0.001^a^

*HD* Huntington’s disease; *FRDA* Friedreich ataxia; *SCA* spinocerebellar ataxia; *PD* Parkinson’s disease; *H&N* head and neck patients; *other* other neurodegenerative diseases; *HC* healthy controls; *yrs* years; speech severity scored on scale of 0–4 where 0 = unremarkable and 4 = severe

^a^H&N vs HC (*p* < 0.001), other diagnosis vs HC (*p* = 0.013)

^b^most groups differed from each other; ^c^one 83-year-old participant received a score of 14 on the MoCA and their data was removed

### Reference tests and clinically meaningful endpoints

All participants were assessed using the Dysarthria Impact Scale (DIS) (index test). Convergent validity was examined by asking participants to also complete the Voice Handicap Index (VHI) [34]. The VHI was used as a reference test for communication performance. The full version of the VHI is a PRO with 30 items exploring the psychosocial consequences of voice disorders. The Short Form 36 (SF-36) [36] was used as a reference test for general health and well being. The SF-36 is a PRO examining generic health and quality of life. The VHI and SF-36 were selected as pragmatic external anchors because no single existing instrument captures dysarthria-related impact across neurological diagnoses; these tools were not treated as gold standards, but as complementary reference measures reflecting related psychosocial and health constructs.

Participants also provided speech samples that were recorded for rating and analysis. Samples were acquired to investigate the link between speech production and self-reported speech-related quality of life. Speech was recorded using the Redenlab® Desktop or Online software in a quiet room. Speech tasks included two iterations of a sustained vowel /a:/ and an unprepared monologue for approximately 1 min. Speech samples were rated independently by two expert speech–language pathologists on an ordinal scale of 0–4 where 0 = unremarkable and 4 = severe. Inter-rater agreement was high (ICC = 0.964 for intelligibility and ICC = 0.958 for naturalness scores) and consistent with prior published protocols using this scale as per previous work [37–39]. Concurrent validity was examined by producing objective composite measures of intelligibility and naturalness using Redenlab’s Analyze^TM^ pipeline derived from the two tasks as per earlier work [40, 41] and comparing them to DIS scores.

### Demonstrating methodological quality of DIS

Although the DIS is not a diagnostic instrument, selected principles from the QUADAS-2 (Quality Assessment of Diagnostic Accuracy Studies-2) framework [42] were used to structure assessment procedures (e.g., blinding, timing of assessments, and reference test independence) to minimize bias and improve transparency. These criteria were considered to minimize bias and improve applicability. The two reference tests, VHI and SF-36, and the index test (DIS), were interpreted separately (i.e., expert clinicians rating speech were blinded to DIS outcomes). The index test was administered to all participants irrespective of clinical presentation. The reference tests were applied to all participants who completed the index test where possible. The duration between the index and reference tests was brief (i.e., within the same testing session or day) to minimize opportunities for participant performance or perception to change between tests (e.g., medication for PD can influence performance over the day). We recruited four different clinical populations to ensure a diverse representative sample of dysarthria was exposed to the index test. Data are presented in detail to adequately describe specific aspects of the index test. The index test can easily be reproduced based on information provided in this manuscript. Lastly, reasonable definitions of normal/abnormal performance on the index and reference tests are provided.

### Statistical analysis

Statistical analyses were conducted using SPSS (IBM SPSS Statistics for Windows, Version 28.0. Armonk, NY: IBM Corp). Validity evidence for the Dysarthria Impact Scale was examined through assessment of convergent validity and discriminative (known-groups) validity. Convergent validity was examined by assessing associations between DIS scores and established patient-reported outcome measures assessing related constructs, including the Voice Handicap Index (VHI) [34] and the SF-36 [36]. Discriminative (known-groups) validity was examined by evaluating the ability of the DIS to differentiate between clinical and control participants using receiver operating characteristic (ROC) analyses, with sensitivity, specificity, and 95% confidence intervals calculated. Cut-off thresholds were chosen as 1 standard deviation below the mean for healthy controls, which gave greater weight to sensitivity, without minimizing the importance of specificity or losing much area under the curve (AUC). Sensitivity scores provided an estimate of the proportion of participants who were identified as presenting with reduced quality of life due to impaired speech. Specificity calculations estimated the proportion of unimpaired participants who were identified as not presenting with reduced QoL. Differences between disease groups were explored with plots and summary statistics. Pearson’s correlations between DIS and clinically relevant metrics such as disease and dysarthria severity and dysarthria were calculated to explore these relationships. Known-groups validity was further examined by comparing DIS performance across neurological diagnostic groups. Internal consistency was evaluated using Cronbach’s alpha for the DIS-22, DIS-17, and DIS-6 total scores in the clinical cohort using complete item responses for each scale. Lower DIS scores indicate worse speech-related quality of life. Cronbach’s alpha was used descriptively to quantify item inter-relatedness and does not establish structural validity.

#### Test–retest reliability

Intra-individual stability and reliability for the DIS were explored by examining agreement between scores provided 1 month apart. Ataxia was selected for test–retest assessment due to its relatively stable short-term clinical profile, allowing evaluation of score stability in the absence of expected clinical change. This short duration was considered long enough to wash out some familiarity effects and brief enough to ensure the disease had not progressed. Agreement was examined using a Bland–Altman plot in addition to correlation, as it was anticipated both samples would be strongly correlated [43]. Only data from the first assessor were included in the final validation data analysis. Repeated assessments were only used for establishing reliability, as determined in our pre-analysis plan.

#### Item reduction

The original list of items in DIS included 22 questions. An iterative approach was adopted to reduce the number of items with the aim of retaining accuracy but reducing completion time and burden on participants. This could then yield a complete set of questions, in addition to a brief version, for use in time-poor clinical settings. At each step, the correlation between the total DIS and each item was calculated, and the item with the weakest correlation (or more than one item if similarly weak) was eliminated (see Supplementary Materials S3). Sensitivity, specificity, and AUC were calculated at each step to help determine the main possibilities for a shorter questionnaire. Principal component analysis (PCA) was initially performed, but resulted in the same item reduction.

#### Calculating minimal detectable change (MDC) and minimal clinically important difference (MCID)

MDC was estimated via within-subject standard deviation (WSSD) values. WSSD was calculated to quantify individual-level test–retest variability for the DIS-17 and DIS-6. The standard deviation of the differences between test and retest scores (SD_diff) was first derived from repeated assessments conducted 1 month apart in the ataxia subgroup. The WSSD was then computed using the standard formula:

$$WSSD = \frac{SDdiff}{{\sqrt 2 }}$$

In the context of test–retest or within-subject variability calculations, the square root of 2 is used to adjust for the variance of the difference, which is twice the within-subject variance.

MCID reflects a patient-perceived threshold of benefit and is ideally estimated using an external anchor to capture what patients judge to be a meaningful change. In this study, MCID was estimated using a distribution-based approach because no anchor (e.g. patient-reported global change) was available. Here we used the widely accepted “0.5 × SD” approach [44], which estimates MCID as half of the standard deviation of the group score distribution.

### Translation

Materials were translated from English into German, French, Polish, Czech, Portuguese, and Turkish (see Supplementary Materials S4 for alternative language versions). Translation protocols are described in Kraus et al. [45] and included both forward and backward translations required for each language.

## Results

Participant groups varied in terms of speech impairment severity, age at assessment, age at onset, disease duration, and MoCA scores (see Table 1).

### Relationship between the dysarthria impact scale and reference tests

The index test (DIS) scores were compared to reference tests (SF-36 and VHI) as well as associated clinical features to examine convergent validity and associations with clinically relevant speech measures. All versions of the DIS (DIS 22-item [DIS-22], DIS 17-item [DIS-17], and DIS 6-item [DIS-6]) were strongly correlated with the reference tests as well as expert blinded consensus ratings of dysarthria (see Table 2 and Supplementary Materials S4 for details). Overall, DIS scores were not associated with disease duration or global cognition, suggesting that DIS responses primarily reflect speech-related impact rather than general disease burden.

**Table 2:** Relationship between DIS-22, DIS-17, and DIS-6 and reference tests

Tool	VHI	SF-36	Perceptual rating of intelligibility	Perceptual rating of naturalness	Disease duration	MoCA^1^	DIS-17	DIS-6	Intelligibility rating (DME)	Naturalness rating (DME)
DIS-22	−0.85 (*p* < 0.001)	0.72 (*p* < 0.001)	−0.75 (*p* < 0.001)	−0.77 (*p* < 0.001)	−0.071 (*p* = .503)	0.34 (*p* < 0.001)	0.996 (*p* < .001)	0.97 (*p* < 0.001)	–	–
DIS-17	−0.85 (*p* < 0.001)	0.711 (*p* < 0.001)	0.78 (*p* < 0.001)	0.79 (*p* < 0.001)	−0.04 (*p* = 0.72)	0.33 (*p* < 0.001)	1.0	0.97 (*p* < 0.001)	0.78 (*p* < 0.001)	0.79 (*p* < 0.001)
DIS-6	−0.8 (*p* < 0.001)	0.68 (*p* < 0.001)	0.78 (*p* < 0.001)	0.79 (*p* < 0.001)	−0.00 (*p* = 0.977)	0.34 (*p* < 0.001)	0.98 (*p* < 0.001)	1.0	0.76 (*p* < 0.001)	0.77 (*p* < 0.001)

*r* Pearson correlation coefficient (2-tailed significance)

*DME* direct magnitude estimation.

^1^MoCA correlations varied across disease groups, i.e., ataxia (DIS-22 *r* = 0.38, DIS-17 *r* = 0.34, DIS-6 *r* = 0.36), HD (DIS-22 p = 0.03, DIS-17 p = 0.03, DIS-6 p = 0.03), PD (DIS-22 p = 0.84, DIS-17 p = 0.71, DIS-6 p = 0.63), head and neck (DIS-22 *p* = 0.81, DIS-17 *p* = 0.76, DIS-6 *p* = 0.77) and healthy controls (DIS-22 *p* = 0.22, DIS-17 *p* = 0.21, DIS-6 *p* = 0.05). Disease duration did not correlate with SF-36, VHI, or MoCA (*P* > 0.01).

Intra-class correlation between perceptual raters was 0.964 for intelligibility and 0.958 for naturalness

### Item reduction

The 22-item DIS was tested and validated in the entire cohort (all participants). Two shorter versions of the DIS were produced following item reduction. Following the removal of redundant items based on statistical item-by-item comparisons, the full version of DIS contained 17 items from the original 22 items. A six-item brief version of the DIS (DIS-6) was also developed using the iterative correlation approach described in the ‘item reduction’ section in the methods. See Supplementary Materials (S3) for a breakdown of the iterative item reduction rounds and corresponding AUCs. Both revised versions of the DIS and their translations into Spanish, French, German, Polish, Czech, and Turkish are available in the Supplementary Materials (S4).

### Sensitivity and specificity analysis

**Fig. 1:**
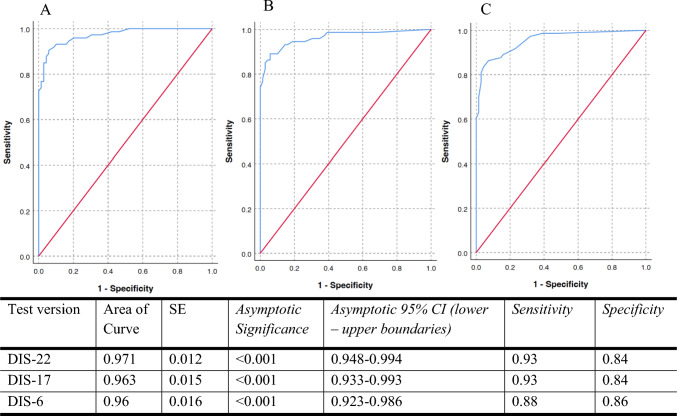
ROC curves for DIS versions with corresponding area under the curve, sensitivity, and specificity of DIS versions. **A** DIS-22; **B** DIS-17; **C** DIS-6

The sensitivity and specificity of three versions of the DIS (DIS-22, DIS-17, DIS-6) are described in Fig. 1. DIS-22 and DIS-17 yielded very similar AUC with equivalent sensitivity and specificity as described in the Statistical analysis section of the methods (i.e., cutoff thresholds were chosen as 1 standard deviation below the mean for healthy controls). The shortest version of DIS (DIS-6) yields 5% less sensitivity than the longer versions (0.93–0.88 = 0.05), potentially not correctly identifying 5 people in 100 with speech-related quality of life issues. It, however, has 2% higher specificity than longer versions of the questionnaire.

### Performance of clinical and control groups on DIS-6 and DIS-17

A test of between-subject effects using univariate analysis of variance revealed a difference across diseases for the DIS-17 (*F* = 47.71, *p* < 0.001) and the DIS-6 (*F* = 42.57, *p* < 0.001) (see Table 3), indicating that both versions of the DIS can capture (i.e., are sufficiently sensitive to) differences in speech-related quality of life resulting from diverse disease groups. In the present cohort, ataxia and Parkinson’s disease groups demonstrated lower speech-related quality of life compared to Huntington’s disease and healthy controls. Head and neck cancer participants functioned as a structural dysarthria comparator group, enabling evaluation of whether DIS performance generalized beyond neurodegenerative etiologies. Their intermediate scores supported the scale’s cross-etiology sensitivity. A post hoc analysis was run comparing the VHI and DIS-6 and DIS-17 using ANOVA with Diagnosis as the predictive factor. VHI does not appear to distinguish between HD and PD, whereas the DIS places HD tangibly (and significantly) higher as estimated by F scores (See Supplementary Materials Table S6).

**Table 3:** DIS scores for full and brief versions across disease groups

Diagnosis		DIS-6 (Min=6, max=30)	DIS-17 (Min=17, max=85)
	N	Mean ± SE (95% CI)	Mean ± SE (95% CI)
Ataxia^a^	73	16.3 ± 0.71 (14.91-17.7)	47.7 ± 1.76 (44.22-51.17)
Huntington’s disease	45	23.87 ± 0.85 (22.2-25.53)	67.98 ± 2.11 (63.85-72.14)
Parkinson’s disease	31	18.1 ± 1.02 (16.09-20.11)	53.61 ± 2.54 (48.6-58.62)
Head and neck ward	20	20.5 ± 1.27 (18.0-23.0)	63.4 ± 3.17 (57.16-69.64)
Healthy controls	69	28.11 ± 0.69 (26.74-29.47)	79.01 ± 1.73 (75.6-82.41)

A^a^ Ataxia diagnostic group includes Autosomal recessive spastic ataxia of Charlevoix-Saguenay (ARSACS), Multiple system atrophy type C (MSA-C), Paraneoplastic cerebellar ataxia, Cerebellar Ataxia with Neuropathy and Vestibular Areflexia Syndrome (CANVAS), Idiopathic ataxia; SE= standard error; CI= confidence interval; Min=minimum; Max=maximum

### Test–retest reliability

**Fig. 2:**
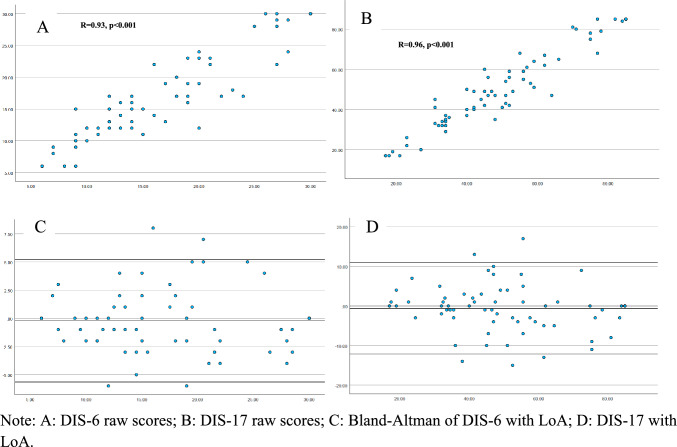
Test–retest for DIS-6 and DIS-17 (1 month apart). **A** DIS-6 raw scores; **B** DIS-17 raw scores; **C** Bland-Altman of DIS-6 with LoA; **D** DIS-17 with LoA

DIS was repeated in the same group of participants (*N* = 71, ataxia only) 1 month apart. Test–retest scores are presented in Fig. 2. A one-sample t-test yielded a mean difference between DIS-17 iterations of -0.62 (*t* = -0.9, *p* = 0.38) and -0.23 (*t* = *-0.69,* *p* = 0.49) for DIS-6, both suggesting agreement between means. The mean difference and standard deviation of each version of DIS were used to construct the limits of agreement (LoA) for Bland–Altman plots [i.e., DIS-6 = -0.23 (mean difference) ± 1.96 (95% of differences) *2.76 (standard deviation) = -5.63/5.27 and DIS-17 = -0.62 ± 1.96*5.88 = -12.15/10.91]; see Fig. 2. Approximately, 5% of responses are expected to fall outside the limits of agreement. Data suggest approximately 7% of responses in this sample are outside of the LoA. Given the retest challenge was only completed by people with ataxia, where disease is known to vary as a function of fatigue and sleep, mood, and physical conditioning, the level of agreement observed here is considered satisfactory. Correlation between both versions was high (*R* = 0.98, *p* < 0.001).

### Internal consistency

Internal consistency was excellent for the DIS-22 (Cronbach’s *α* = 0.970; *N* = 169 complete cases) and DIS-17 (*α* = 0.968; *N* = 169) in the clinical cohort, and remained high for the DIS-6 short form (*α* = 0.917; *N* = 174) (see Supplementary Materials Table S7 for details).

### MDC and MCID

Using the reported standard deviation of the difference scores, the WSSD for both the DIS-17 and DIS-6 was calculated as follows: SD_diff is the standard deviation of the differences between test and retest scores (√2 ≈ 1.414), DIS-17: SD_diff = 5.88 [WSSD = 5.88/1.414 ≈ 4.16] and DIS-6: SD_diff = 2.76 [WSSD = 2.76/1.414 ≈ 1.95]. WSSD is used here as a measure of minimal detectable change.

MCIDs reflect a distribution-based approximation of change magnitude that may be considered meaningful, pending anchor-based confirmation. The estimated minimal clinically important differences (MCIDs) for DIS-6 and DIS-17 ranged from approximately 5 to 15 points across diagnostic groups, providing provisional distribution-based thresholds to assist interpretation of change, pending confirmation using anchor-based or longitudinal methods (Supplementary Materials S5).

## Discussion

The Dysarthria Impact Scale is a brief, repeatable and validated patient-reported outcome for measuring the effect of neurological speech impairment on the speaker. It yields meaningful data on dysarthria in people with ataxia, Parkinson’s disease, Huntington’s disease and head and neck patients. The DIS is intended as a patient-reported outcome measure for monitoring perceived impact and change over time at the group or individual level. It is not designed as a diagnostic tool nor as a standalone measure of disease severity. The DIS’ utility was established using published guidance on clinical tool development, expert judgment, clinical experience, published reports and familiarity with legacy tools in the domain. It provides a sensitive and specific clinical outcome assessment that can be administered as part of experimental and clinical assessment and trial protocols, translated into six languages.

Historical PROs for motor speech are limited by their length (e.g., up to 160 items), complexity (e.g., positive and negative wording), limited validation data, or validation in non-dysarthria contexts (e.g., voice only). The DIS contains questions related to physical, emotional, and functional performance, focused on practical scenarios where speech may impact interactions (e.g., social isolation, talking on the phone, complex conversations), supporting content validity of the scale.

Speech-related quality of life as measured by both the full and short forms of the DIS were strongly linked to overall dysarthria severity, as measured by expert speech pathologist ratings, indicating strong content and concurrent validity. In the present cohort, greater dysarthria severity was associated with lower speech-related quality of life. DIS scores were also aligned with performance on the general health questionnaire (SF-36) and the voice-related quality of life PRO (VHI), thus indicating good convergent validity. Cognition, as measured by the MoCA, had a small influence on responses to DIS. Scores on the DIS were not directly related to disease duration in these cohorts but did vary as a function of disease itself, indicating good known-groups validity. These data suggest that cognition and overall disease duration may contribute to QoL but are not primary drivers related to communication. Consideration of speech as a distinct domain of assessment will enable more accurate phenotyping and acknowledgment of the relative contributions speech deficits can make to a patients’ profile.

The DIS demonstrated strong convergent validity with both the VHI (*r* = –0.85 for DIS-22 and DIS-17; –0.80 for DIS-6) and the SF-36 (*r* = 0.72 for DIS-22; 0.68 for DIS-6). These robust correlations support the DIS as a valid measure of speech-related quality of life, capturing overlapping constructs of vocal handicap and general health-related quality of life. The consistency across short and long versions reinforces its applicability in time-constrained settings. However, convergent validity may be limited by the VHI’s focus on voice rather than articulatory impairment and the SF-36’s lack of communication-specific domains. Structural validity and hypothesis-driven construct validation were not formally assessed in this study, reflecting the limited availability of speech-specific patient-reported outcome measures applicable across neurological diagnoses.

Perhaps surprisingly, DIS scores were not associated with disease duration (DIS-22: *r* = –0.071, *p* = 0.503) or global cognitive function (MoCA correlations nonsignificant in most disease groups), suggesting that the tool discriminates between speech-specific impairment and other disease features. This suggests that the DIS captures speech-specific impact rather than broader disease characteristics. However, the small but significant MoCA correlations in some subgroups (e.g., HD) suggest a potential cognitive bias in self-report that warrants further investigation in populations with more pronounced cognitive decline [46].

The test–retest reliability of the DIS was high across a 1-month interval (*r* = 0.98 for both DIS-6 and DIS-17), indicating excellent temporal stability. Bland–Altman analysis revealed acceptable limits of agreement (DIS-6: –5.63 to 5.27; DIS-17: –12.15 to 10.91), with 7% of scores falling outside these bounds. Given that this was assessed in a population (ataxia) with known day-to-day speech variability [47], the observed variability is considered acceptable. Future work is needed to confirm these findings across other clinical groups and longer periods.

The DIS-22 and DIS-17 exhibited comparable sensitivity (0.93) and specificity (0.84), with negligible loss of AUC (DIS-22: 0.971 vs DIS-17: 0.963). The DIS-6 retained a high AUC (0.96) with slightly reduced sensitivity (0.88) but increased specificity (0.86), suggesting it may be preferable in screening scenarios where false positives are more problematic. The minor reduction in sensitivity for DIS-6 likely reflects exclusion of items relating to complex emotional or interactional speech tasks, which may be more affected in early-stage or mild cases.

The minimal clinically important difference was not calculated using direct patient-reported anchors (such as patient global impression of change). Instead, a distribution-based method was applied to estimate a provisional MCID. According to this method, the MCID was estimated as half the standard deviation of baseline scores across clinical cohorts.

This approach provides a practical and provisional clinical threshold for interpreting changes on the DIS, pending future confirmation using patient-reported anchors or longitudinal intervention studies. Comparison of within-subject standard deviation (WSSD) and minimal clinically important difference (MCID) values across diagnostic groups provides critical insight into the interpretability of score changes on the DIS. For both DIS-6 and DIS-17, WSSD values were consistently lower than corresponding MCID estimates across all disease groups, indicating that typical intra-individual variability is unlikely to obscure potentially meaningful changes, pending anchor-based confirmation. This separation between measurement noise (WSSD) and meaningful change (MCID) supports the DIS as a stable tool with potential utility for monitoring treatment effects or disease progression at a group level, pending longitudinal validation. Interestingly, WSSD values were similar across groups, whereas MCID values were more variable, reflecting differences in overall speech-related burden and score dispersion between conditions. For example, ataxia and Parkinson’s disease groups exhibited larger MCID values, consistent with broader variability in speech impact severity. These findings suggest that while the DIS performs robustly across populations, disease-specific benchmarks may enhance interpretability in longitudinal applications.

At a group level, mean QoL scores varied significantly across groups for both DIS-6 and DIS-17 (*p* < 0.001). Ataxia and Parkinson’s disease groups had the lowest scores (DIS-17 means: 47.7 and 53.6), indicating more impaired speech-related quality of life, aligning with speech severity observed in the present cohort [37–40, 48, 49]. In contrast, HD participants reported comparatively higher DIS scores (mean = 68.0), despite known speech changes in HD [8]. This may reflect reduced insight, anosognosia, or distinct communicative priorities [50]. Head and neck cancer patients had intermediate scores as would be expected in wards where approximately one-third of patients present dysarthria [51], with supporting the scale’s sensitivity across etiologies. Healthy controls scored near the ceiling, confirming discriminative capability.

### Limitations

The DIS has strong psychometric properties; however, this study has several limitations that warrant consideration. First, the cross-sectional design precludes conclusions about the scale’s responsiveness to disease progression or intervention. While short-term test–retest reliability was established over a 1-month interval in individuals with ataxia, longer-term data are needed to assess whether the DIS can reliably detect clinically meaningful change over time. The absence of longitudinal data also limits the ability to derive robust estimates of minimal clinically important differences. Additionally, the analysis of group differences may be constrained by unequal sample sizes across diagnostic categories, which may influence statistical power and generalisability of findings. Sensitivity analyses excluding the head and neck cancer group did not materially alter overall validity estimates (data not shown), supporting their inclusion as a comparator cohort without biasing primary neurological validity outcomes. Test–retest reliability was limited to a single clinical group, further constraining inferences about stability across broader populations. While cognitive screening using the MoCA suggested minimal influence of cognition on DIS scores overall, subgroup analyses indicated mild associations in some groups, such as Huntington’s disease. This may reflect reduced insight or executive dysfunction, which could affect the reliability of self-reported outcomes. While the DIS was translated into multiple languages, psychometric evaluation was restricted to the English and German versions; further validation is required to confirm cross-cultural and linguistic equivalence. Patient involvement during item development was limited to a small purposive sample of individuals with Friedreich ataxia. While this ensured relevance for progressive dysarthria, broader engagement across diagnostic groups was not undertaken and may have resulted in omission of condition-specific concerns. Expanded patient co-design across additional neurological conditions represents an important priority for future refinement. Finally, the use of reference tools such as the SF-36 and VHI, though appropriate, may not fully capture the multidimensional nature of communication-related quality of life, highlighting the need for additional anchors (e.g., patient global impression of change) in future work. Anchor-based MCID estimation using patient-reported global change was not undertaken in this study and represents an important priority for future validation work.

## Conclusions

Motor speech disorders can lead to dramatic reductions in quality of life [9, 52, 53] and quality of disease management [54]. The burden of communication decline is dealt to the affected individual, their entire family and wider network [55–58]. Impaired speech leads to daily disadvantage, triggering social marginalization and underemployment [3]. The DIS can quantify the deleterious consequences of dysarthria including limited activities and social participation (e.g. communicating over the telephone, talking to friends), and that these are related to key environmental factors (e.g. communicating in noisy environments). The magnitude of dysarthria’s impact on the speaker and their community means it is essential that speech-related quality of life is quantified in clinical and experimental settings.

The DIS is a brief, reliable, and valid patient-reported outcome measure that captures the impact of speech impairment on quality of life across diverse neurological conditions. It demonstrates strong convergent and discriminative validity, excellent test–retest reliability, and robust sensitivity and specificity across full and abbreviated versions. The tool distinguishes between disease groups and aligns closely with perceptual ratings of dysarthria severity, supporting its utility in clinical assessment and research settings, with further longitudinal validation required before routine use as a clinical trial end point. While further longitudinal validation and cross-cultural testing are needed, the DIS represents a clinically meaningful and scalable instrument for assessing speech-related quality of life in individuals with motor speech disorders.

## Electronic supplementary material

Below is the link to the electronic supplementary material.

Supplementary material 1 (DOCX 889 kb)

## Data Availability

Data not shared due to ethical/privacy concerns. Access possible via Data Use Agreement (contact Prof Adam Vogel, vogela@unimelb.edu.au).
