# Supplementary material for: Development and validation of the dysarthria impact scale: a patient-reported outcome for motor speech disorders
Source: J Neurol. 2026 Mar 10;273(3):195. doi: 10.1007/s00415-026-13740-1 (PMC12975812; doi:10.1007/s00415-026-13740-1)
Supplement: Supplementary file 1 — Supplementary material 1 (DOCX 889 kb) [file 415_2026_13740_MOESM1_ESM.docx]

**Supplementary Materials**

**Development and validation of the Dysarthria Impact Scale**

Adam P. Vogel PhD ^1,2,3^, Lisa Graf MSc ^2,3^, Merit Weiß MD ^2^, Cheuk S. J. Chan PhD ^1^, Graham Hepworth PhD ^4^, Matthis Synofzik MD ^2,5^

1. Department of Neurodegenerative Diseases, Hertie-Institute for Clinical Brain Research and Center of Neurology, University of Tübingen, Germany

2. Speech Pathology, School of Health Sciences, The University of Melbourne, Australia

3. Redenlab Ltd., Melbourne, Australia

4. Statistical Consulting Centre, The University of Melbourne, Melbourne, Australia

5. German Center of Neurodegenerative Diseases (DZNE), Tübingen, Germany

**Table S1 Tools identified during the review of existing resources**

| **Instrument** | **Construct / focus** | **Domain/s** | **Respondent/s** | **Target population / condition** | **Format / length** | **Psychometric highlights** |
| --- | --- | --- | --- | --- | --- | --- |
| Communication Outcomes After Stroke (COAST) ^1^ | Communication activity, participation, and QoL | Participation, Emotional, Social | Self-report (aphasia-accessible); proxy version available | Stroke (aphasia and/or dysarthria) | 20–25 items (VAS) | Good internal consistency (α≈0.85); test–retest reliability |
| Communicative Participation Item Bank (CPIB / CPIB-D) ^2^ | Communicative participation restrictions | Participation, Social, Psychosocial | Self-report (adaptive electronic or interviewer-assisted) | Voice and motor speech disorders (ALS, PD, stroke, head & neck cancer) | Item bank (~10 items short form) | Rasch-validated; high reliability; cross-disorder comparability |
| Dysarthria Impact Profile (DIP) ^3^ | Psychosocial impact of acquired dysarthria | Psychosocial, Emotional, Self-concept, Communication participation | Self-report (clinician may facilitate) | Acquired dysarthria (stroke, PD, MS, TBI) | 48 items, 5 sections (A–E) | Good internal consistency (α > 0.8); strong intra-rater reliability; convergent validity with HISD-II |
| Glottal Function Index (GFI) ^4^ | Perceived glottal effort/strain | Physical, Functional | Self-report | General voice disorders | 4 items | Validated for screening; good reliability |
| Overall Assessment of Speaker’s Experience of Stuttering (OASES) ^5^ | Stuttering impact across life areas | Emotional, Cognitive, Social participation, Functional | Self-report | Adults/adolescents who stutter | 4 sections (~100 items) | Excellent validity and reliability (α > 0.9); sensitive to therapy |
| Quality of Life in Dysarthria (QOL-DyS) ^6^ | QoL and psychosocial burden in dysarthria | Physical, Psychosocial, Emotional | Self-report | Dysarthria due to stroke or ALS | Likert (paper) | Limited validation; low evidence quality |
| Vocal Fatigue Index (VFI) ^7^ | Vocal effort, fatigue, recovery | Physical, Functional | Self-report | Professional and occupational voice users | 19 items | Excellent internal consistency (α = 0.94); validated factors |
| Voice Handicap Index (VHI) ^8^ | Voice-related handicap and QoL impact | Physical, Functional, Emotional | Self-report | Adults with voice disorders | 30 items, 5-point Likert | Excellent reliability (α > 0.9); convergent validity with V-RQOL |
| Voice Handicap Index-10 (VHI-10) ^9^ | Brief version of VHI | Physical, Functional, Emotional | Self-report | Adults with voice disorders | 10 items | Strong internal consistency (α≈0.95); high responsiveness to therapy |
| Voice Outcome Survey (VOS) ^10^ | Functional impact post-thyroidectomy | Functional, Physical | Self-report | Post-thyroidectomy, laryngeal surgery | 5 items | Sensitive to surgical improvement; acceptable reliability |
| Voice-Related Quality of Life (V-RQOL) ^11^ | QoL impact of voice | Physical, Social, Emotional | Self-report | Voice disorders | 10 items | High reliability; strong construct validity |

**S2 Consumer feedback on communication relevant domains**

Consumers were asked if, where and when they had trouble communicating because of their speech, without any pre-determined scenarios or examples provided to respondents. They were then asked how this made them feel. Answers not directly relevant to the focus of speech were not included.

Responses are summarised below:

- Locations and situations where speech impacted communication;
  - Talking in loud environments, e.g. café, pub, shopping malls/centers
    - Having difficulty being heard
    - Difficulty hearing others
  - During phone calls
  - Mealtimes, at the dinner table
  - At work
- Respondents felt that people familiar with them could be annoyed, frustrated.
- A perception that people unfamiliar to respondents assumed they have a hearing impairment, are ‘stupid’, there is ‘something wrong with me’, are ‘slow’, ‘thick’, or ‘drunk’.
- ‘Most people don’t say anything’, but occasionally when on the phone people will ‘ask what is wrong with me’.

**S3 Domain selection**

**Chosen domains and items**

Exemplar questions were designed to capture information across the select domains. Table S3.1 shows the first round of domains and items selected for interrogation. Successive iterations are described in Tables S3.2-3.4, showing the working progress across domains and comments made by the investigator team. Item refinement occurred following discussion among the assembled research team. Item reduction continued via statistical methods to remove redundant questions after the larger survey was applied to clinical respondents and controls (S4).

Literature (S1), consumer responses (S2) and clinical experience informed selection of domains relevant to the impact of dysarthria on quality life. Specific themes included functional (e,g., as used in the Voice Handicap Index (VHI)^8^), social-emotional and physical functioning (e.g., as used in the Voice Related Quality of Life (VRQOL)^11^, and acceptance (e.g., as used in the Dysarthria Impact Profile (DIP)^3^). Using this information, the lead author created questions across the three domains of functional, emotional and physical for the first iteration.

**Table S3.1** *Iteration 1*

| **Functional** | **Emotional** | **Physical** |
| --- | --- | --- |
| 1. noisy space 2. providing information 3. describing events 4. turn taking 5. multi-speaker setting 6. unfamiliar listeners 7. asked for repetition 8. reduction in content 9. avoid talking on the phone 10. not participating in complex or quick conversations 11. find alternatives for communication, e.g., text, IM, email | 1. social setting 2. workplace 3. interacting with friends, family self-conscious 4. avoidance talking 5. dependence on others 6. frustration 7. isolation / loneliness 8. treat me differently 9. respond to me differently 10. self-esteem / confidence 11. less competent 12. change in identity 13. stigma 14. condescension from listener 15. embarrassment | 1. dysarthria severity 🡺 better: dysarthria intelligibility? 2. speech is different at different times in the day e.g., when tired 3. increased effort to be understood |

All authors then reviewed the items from iteration one. Items deemed duplicative or unnecessary were highlighted and comments added to the version.

**Table S3.2** *Iteration 2*

| **Functional** | **Emotional** | **Physical** |
| --- | --- | --- |
| 1. noisy space 2. providing information 🡺 replace with Q3 3. describing events 4. turn taking 5. multiple speaker setting 🡺 same/like Q4 and Q10 6. unfamiliar listeners 7. asked for repetition 8. reduction in content 9. avoid talking on the phone 10. not participating in complex or quick conversations 11. find alternatives for communication, e.g., text, IM, email | 1. social setting 2. workplace 3. interacting with friends, family self-conscious 4. avoidance talking 🡺 is already part of Q8, Q9, Q14 5. dependence on others 6. frustration 7. isolation / loneliness 8. treat me differently 🡺 is the same/like Q20, merge with Q20 9. respond to me differently 10. self-esteem / confidence 11. less competent 12. change in identity 🡺 like Q21 13. stigma 14. condescension from listener 🡺 is the same/like Q20, merge with Q20 15. embarrassment 🡺 is like Q21, merge with Q21 | 1. dysarthria severity 🡺 better: dysarthria intelligibility? 2. speech is different at different times in the day e.g., when tired 3. increased effort to be understood |

At a follow up meeting, The same process was followed one month later for the

**Table S3.3** *Iteration 3*

| **Functional** | **Emotional** | **Physical** |
| --- | --- | --- |
| 1. noisy space 2. providing information 🡺 replace with Q3 AGREED 3. describing events 4. turn taking 5. multiple speaker setting 🡺 same/like Q4 and Q10 DISAGREE ON BOTH 6. unfamiliar listeners 7. asked for repetition 8. reduction in content 9. avoid talking on the phone 10. not participating in complex or quick conversations 11. find alternatives for communication, e.g., text, IM, email | 1. social setting 2. workplace 3. interacting with friends, family self-conscious 4. avoidance talking 🡺 is already part of Q8 NOPE, Q9 YES IN PART – SPECIFIC SETTING, Q14 SAME AS PREVIOUS POINT 5. dependence on others 6. frustration 7. isolation / loneliness 8. treat me differently 🡺 is the same as Q20, merge with Q20 AGREED 9. respond to me differently 10. self-esteem / confidence 11. less competent 12. change in identity 🡺 like Q21 PARTLY DISAGREE 13. stigma 14. condescension from listener 🡺 is the same like Q20, merge with Q20 – SORT OF- WE DO HAVE SOME DUPLICATION HERE 15. embarrassment 🡺 is similar to Q21, merge with Q21 CONCEPTUALISED DIFFERENTLY | 1. dysarthria severity 🡺 better: dysarthria intelligibility? 2. speech is different at different times in the day e.g., when tired 3. increased effort to be understood |

The themes and items listed in iteration three were then converted to 22 items for application in the validation component of the study.

**Table S3.4** *Iteration 4*

Table S3.4 documents internal expert discussion during the final thematic consolidation stage. Comments reflect working deliberations prior to item formalisation.

| **Functional** | **Emotional** | **Physical** |
| --- | --- | --- |
| 1. noisy space 2. providing information 🡺 replace with Q3 AGREED 3. describing events 4. turn taking 5. multiple speaker setting 🡺 same/like Q4 and Q10 DISAGREE ON BOTH 6. unfamiliar listeners 7. asked for repetition 8. reduction in content 9. avoid talking on the phone 10. not participating in complex or quick conversations 11. find alternatives for communication, e.g., text, IM, email | 1. social setting 2. workplace 3. interacting with friends, family self-conscious 4. avoidance talking 🡺 is already part of Q8 NOPE, Q9 YES IN PART – SPECIFIC SETTING, Q14 SAME AS PREVIOUS POINT 5. dependence on others 6. frustration 7. isolation / loneliness 8. treat me differently 🡺 is the same as Q20, merge with Q20 AGREED 9. respond to me differently 10. self-esteem / confidence 11. less competent 12. change in identity 🡺 like Q21 PARTLY DISAGREE 13. stigma 14. condescension from listener 🡺 is the same like Q20, merge with Q20 – SORT OF- WE DO HAVE SOME DUPLICATION HERE 15. embarrassment 🡺 is similar to Q21, merge with Q21 CONCEPTUALISED DIFFERENTLY | 1. dysarthria severity 🡺 better: dysarthria intelligibility? 2. speech is different at different times in the day e.g., when tired 3. increased effort to be understood |

**Table S3.5. Item retention across Dysarthria Impact Scale development and reduction stages**

Themes and candidate concepts identified through literature review, consumer feedback, and expert discussion across Iterations 1–4 (Tables S3.1–S3.4) were consolidated into 22 formal items following Iteration 4. Table S3.5 maps these items to their primary source themes and documents retention across subsequent development and reduction stages.

| **DIS Item** | **Brief item descriptor** | **Domain** | **Primary source theme (thematic development prior to item formalisation)** | **Derived from thematic pool (S3.1–S3.4)** | **Post-expert review** | **DIS-22** | **DIS-17** | **DIS-6** |
| --- | --- | --- | --- | --- | --- | --- | --- | --- |
| dis_1 | Speech effort | Physical | Effort required to speak; physical strain during speech | ✓ | ✓ | ✓ | ✓ |  |
| dis_2 | Being understood | Functional | Being understood by others; need to repeat oneself | ✓ | ✓ | ✓ | ✓ | ✓ |
| dis_3 | Speech frustration | Emotional | Frustration and emotional response to speech difficulties | ✓ | ✓ | ✓ |  | ✓ |
| dis_4 | Speech fatigue | Physical | Speech-related fatigue and reduced stamina | ✓ | ✓ | ✓ | ✓ |  |
| dis_5 | Avoiding conversations | Emotional | Avoidance of speaking situations due to speech difficulty | ✓ | ✓ | ✓ |  |  |
| dis_6 | Speaking in groups | Functional | Difficulty communicating in group or noisy environments | ✓ | ✓ | ✓ |  |  |
| dis_7 | Telephone communication | Functional | Problems using the telephone or remote communication | ✓ | ✓ | ✓ | ✓ |  |
| dis_8 | Speech confidence | Emotional | Reduced confidence when speaking | ✓ | ✓ | ✓ | ✓ |  |
| dis_9 | Social participation | Functional | Impact of speech difficulties on social participation | ✓ | ✓ | ✓ | ✓ |  |
| dis_10 | Speech clarity | Physical | Reduced clarity or intelligibility of speech | ✓ | ✓ | ✓ | ✓ | ✓ |
| dis_11 | Repeating oneself | Functional | Need to repeat or rephrase speech to be understood | ✓ | ✓ | ✓ | ✓ |  |
| dis_12 | Emotional impact of speech | Emotional | Emotional impact of having a speech disorder | ✓ | ✓ | ✓ | ✓ |  |
| dis_13 | Communication independence | Functional | Loss of independence in communication | ✓ | ✓ | ✓ | ✓ |  |
| dis_14 | Speech-related stress | Emotional | Stress or anxiety related to speaking | ✓ | ✓ | ✓ | ✓ |  |
| dis_15 | Day-to-day communication | Functional | Impact of speech difficulties on everyday communication | ✓ | ✓ | ✓ | ✓ | ✓ |
| dis_16 | Speech naturalness | Physical | Changes in speech naturalness or effortfulness | ✓ | ✓ | ✓ | ✓ | ✓ |
| dis_17 | Impact on relationships | Emotional | Impact of speech difficulties on close relationships | ✓ | ✓ | ✓ | ✓ |  |
| dis_18 | Communication participation | Functional | Restrictions in communicative participation | ✓ | ✓ | ✓ | ✓ |  |
| dis_19 | Emotional wellbeing | Emotional | Broader emotional wellbeing affected by speech | ✓ | ✓ | ✓ | ✓ |  |
| dis_20 | Overall speech impact | Functional | Overall perceived impact of speech difficulties | ✓ | ✓ | ✓ | ✓ | ✓ |
| dis_21 | Coping with speech changes | Emotional | Coping with and adapting to speech changes | ✓ | ✓ | ✓ |  |  |
| dis_22 | Speech limitations | Physical | Perceived limitations caused by speech difficulties | ✓ | ✓ | ✓ |  |  |

Items were formalised after thematic development and expert review (Iterations 1–4; Tables S3.1–S3.4). Table S3.5 therefore documents item-level retention from the first itemised version onward, rather than from the initial thematic pool.

Together, Tables S3.1–S3.5 provide a transparent audit trail from thematic development to item-level retention across all DIS versions.

**S4 Steps involved in item removal**

The statistical approach for item removal is described in the Methods section of the manuscript. Item removal was guided by statistical redundancy while ensuring that retained items continued to cover functional, emotional, and physical impact domains identified during earlier qualitative and clinical review. The aim of item removal was to yield the most accurate version of the tool while using the least number of items. Item removal involved discarding more than one item with the weakest correlation.  Below are the steps taken to reduce the DIS from 22 items to 17 and then to 6. Sensitivity and specificity were chosen using mean and standard deviation for the control group as a cutoff.

AUC=Area under the curve; Sens=sensitivity; spec=specificity

All 22 items:       AUC = 0.971        Sens = 0.932       Spec = 0.841

Weakest correlation with item 3 (r = 0.773), item 5 (r = 0.678), item 6 (r = 0.759), item 21 (r = 0.777), item 22 (r = 0.768).

17 items:             AUC = 0.963        Sens = 0.932       Spec = 0.841

Weakest correlation with item 8 (r = 0.800), item 17 (r = 0.797).

15 items:             AUC = 0.967        Sens = 0.932       Spec = 0.826

Weakest correlation with item 1 (r = 0.817), item 12 (r = 0.817), item 13 (r = 0.818).

12 items:             AUC = 0.967        Sens = 0.918       Spec = 0.870

Weakest correlation with item 9 (r = 0.820).

11 items:             AUC = 0.966        Sens = 0.904       Spec = 0.841

Weakest correlation with item 7 (r = 0.842), item 18 (r = 0.845), item 19 (r = 0.849).

8 items:             AUC = 0.964        Sens = 0.877       Spec = 0.841

Weakest correlation with item 11 (r = 0.881), item 14 (r = 0.878).

6 items:             AUC = 0.955        Sens = 0.877       Spec = 0.855

Weakest correlation with item 15 (r = 0.888).

**S4 Dysarthria Impact Scale versions**

**Dysarthria Impact Scale (FULL VERSION) *English***

| How would you rate the overall quality of your speech? | Very good (1) | Good (2) | Fair (3) | Poor (4) | Very poor (5) |
| --- | --- | --- | --- | --- | --- |

Please rate how much you agree with the following statements about your speech from "fully agree" to "do not agree at all."

|  | Fully agree (1) | Agree (2) | Neither agree nor disagree (3) | Do not agree (4) | Do not agree at all (5) |
| --- | --- | --- | --- | --- | --- |
| 1. Because of my speech, I find it difficult to be understood in noisy environments. |  |  |  |  |  |
| 1. My speech affects my social life. |  |  |  |  |  |
| 1. Because of my speech, I cannot communicate to a full extent. |  |  |  |  |  |
| 1. Because of my speech, I avoid talking to strangers. |  |  |  |  |  |
| 1. I feel self-conscious when speaking. |  |  |  |  |  |
| 1. I feel like people treat me differently because of my speech. |  |  |  |  |  |
| 1. People unfamiliar with me have difficulty understanding my speech. |  |  |  |  |  |
| 1. It takes a great deal of effort to make myself understood. |  |  |  |  |  |
| 1. I depend on others because of my speech. |  |  |  |  |  |
| 1. I feel like people consider me less competent because of my speech. |  |  |  |  |  |
| 1. I am frustrated with my speech. |  |  |  |  |  |
| 1. Because of my speech, I avoid talking on the phone. |  |  |  |  |  |
| 1. Because of my speech, it is difficult for me to describe events. |  |  |  |  |  |
| 1. I feel less competent because of my speech. |  |  |  |  |  |
| 1. I am often asked to repeat myself. |  |  |  |  |  |
| 1. My speech affects my ability to perform in the workplace. * |  |  |  |  |  |
| 1. Because of my speech, I have difficulty participating in fast or complex conversations. |  |  |  |  |  |

**In non-working participants: does their speech affect communication in a bureaucratic/business environment? e.g. banking, official necessities. ©️ 2016-2026 Vogel, Graf, Synofzik*

**Dysarthria Impact Scale (DIS-6) *English***

| How would you rate the overall quality of your speech? | Very good (1) | Good (2) | Fair (3) | Poor (4) | Very poor (5) |
| --- | --- | --- | --- | --- | --- |

Please rate how much you agree with the following statements about your speech from "fully agree" to "do not agree at all."

|  | Fully agree (1) | Agree (2) | Neither agree nor disagree (3) | Do not agree (4) | Do not agree at all (5) |
| --- | --- | --- | --- | --- | --- |
| 1. My speech affects my social life. |  |  |  |  |  |
| 1. I feel isolated or lonely because of my speech. |  |  |  |  |  |
| 1. People unfamiliar with me have difficulty understanding my speech. |  |  |  |  |  |
| 1. Because of my speech, I avoid talking on the phone. |  |  |  |  |  |
| 1. Because of my speech, it is difficult for me to describe events. |  |  |  |  |  |
| 1. Because of my speech, I have difficulty participating in fast or complex conversations. |  |  |  |  |  |

*©️ 2016-2026 Vogel, Graf, Synofzik*

**Dysarthria Impact Scale (FULL VERSION) *German, “Dysarthrie-Auswirkungsskala”***

| Wie beurteilen Sie insgesamt die Qualität Ihres Sprechens? | Sehr gut (1) | Gut (2) | In Ordnung (3) | Schlecht (4) | Sehr schlecht (5) |
| --- | --- | --- | --- | --- | --- |

Bitte bewerten Sie, wie sehr Sie den folgenden Aussagen über Ihre Sprache zustimmen, von "stimme vollständig zu" bis "stimme überhaupt nicht zu".

|  | Stimme vollständig zu (1) | Stimme zu (2) | Ich bin mir nicht sicher (ob ich zustimme oder nicht) (3) | Stimme nicht zu (4) | Stimme überhaupt nicht zu (5) |
| --- | --- | --- | --- | --- | --- |
| 1. Aufgrund meines Sprechens finde ich es schwer, in lauter Umgebung verstanden zu werden. |  |  |  |  |  |
| 1. Mein Sprechen beeinträchtigt mein Sozialleben. |  |  |  |  |  |
| 1. Aufgrund meines Sprechens kann ich Inhalte nicht in vollem Ausmaß kommunizieren. |  |  |  |  |  |
| 1. Aufgrund meines Sprechens vermeide ich es, mit Fremden zu reden. |  |  |  |  |  |
| 1. Ich fühle mich unsicher, wenn ich spreche. |  |  |  |  |  |
| 1. Ich glaube, dass man mich wegen meines Sprechens anders behandelt. |  |  |  |  |  |
| 1. Menschen, die mich nicht gut kennen, verstehen mich schlecht. |  |  |  |  |  |
| 1. Es kostet mich viel Mühe, verstanden zu werden. |  |  |  |  |  |
| 1. Aufgrund meines Sprechens bin ich von anderen abhängig. |  |  |  |  |  |
| 1. Ich glaube, dass andere Menschen mich aufgrund meines Sprechens für weniger qualifiziert halten. |  |  |  |  |  |
| 1. Mein Sprechen frustriert mich. |  |  |  |  |  |
| 1. Aufgrund meines Sprechens vermeide ich es, zu telefonieren. |  |  |  |  |  |
| 1. Aufgrund meines Sprechens fällt es mir schwer, Ereignisse zu beschreiben. |  |  |  |  |  |
| 1. Aufgrund meines Sprechens fühle ich mich weniger kompetent. |  |  |  |  |  |
| 1. Ich werde oft gebeten, etwas zu wiederholen. |  |  |  |  |  |
| 1. Mein Sprechen beeinträchtigt meine Leistung am Arbeitsplatz. * |  |  |  |  |  |
| 1. Aufgrund meines Sprechens fällt es mir schwer, an komplexen oder schnellen Unterhaltungen teilzunehmen. |  |  |  |  |  |

**Bei nicht erwerbstätigen Personen: Beeinträchtigung der Kommunikation in geschäftlichem Umfeld? (z.B. Behörden, Bankgeschäfte etc.) ©️ 2016-2026 Vogel, Graf, Synofzik*

**Dysarthria Impact Scale (DIS-6) *German, “Dysarthrie-Auswirkungsskala”***

| Wie beurteilen Sie insgesamt die Qualität Ihres Sprechens? | Sehr gut (1) | Gut (2) | In Ordnung (3) | Schlecht (4) | Sehr schlecht (5) |
| --- | --- | --- | --- | --- | --- |

Bitte bewerten Sie, wie sehr Sie den folgenden Aussagen über Ihre Sprache zustimmen, von "stimme vollständig zu" bis "stimme überhaupt nicht zu".

|  | Stimme vollständig zu (1) | Stimme zu (2) | Ich bin mir nicht sicher (ob ich zustimme oder nicht) (3) | Stimme nicht zu (4) | Stimme überhaupt nicht zu (5) |
| --- | --- | --- | --- | --- | --- |
| 1. Mein Sprechen beeinträchtigt mein Sozialleben. |  |  |  |  |  |
| 1. Aufgrund meines Sprechens fühle ich mich einsam oder isoliert. |  |  |  |  |  |
| 1. Menschen, die mich nicht gut kennen, verstehen mich schlecht. |  |  |  |  |  |
| 1. Aufgrund meines Sprechens vermeide ich es, zu telefonieren. |  |  |  |  |  |
| 1. Aufgrund meines Sprechens fällt es mir schwer, Ereignisse zu beschreiben. |  |  |  |  |  |
| 1. Aufgrund meines Sprechens fällt es mir schwer, an komplexen oder schnellen Unterhaltungen teilzunehmen. |  |  |  |  |  |

*©️ 2016-2026 Vogel, Graf, Synofzik*

**Dysarthria Impact Scale (FULL VERSION) *Portuguese, “Escala de impacto da disartria”***

| Qual você diria que é a qualidade geral da sua fala? | Muito boa (1) | Boa (2) | Moderada (3) | Ruim (4) | Muito ruim (5) |
| --- | --- | --- | --- | --- | --- |

Por favor, assinale (faça um ‘x’) o quanto você concorda com cada frase abaixo, desde “Concordo totalmente” até “Não concordo nem um pouco”.

|  | Concordo totalmente (1) | Concordo (2) | Nem concordo, nem discordo (3) | Não concordo muito (4) | Não concordo nem um pouco (5) |
| --- | --- | --- | --- | --- | --- |
| 1. É difícil entender a minha fala em ambientes ruidosos ou barulhentos. |  |  |  |  |  |
| 1. Minha fala afeta minha vida social. |  |  |  |  |  |
| 1. Eu não consigo me comunicar plenamente por causa da minha fala. |  |  |  |  |  |
| 1. Eu evito falar com estranhos por causa da minha fala. |  |  |  |  |  |
| 1. Eu fico com vergonha ao falar. |  |  |  |  |  |
| 1. Eu sinto que as pessoas me tratam de maneira diferente por causa da minha fala. |  |  |  |  |  |
| 1. Pessoas que não me conhecem bem tem dificuldade em entender o que eu falo. |  |  |  |  |  |
| 1. Eu preciso me esforçar bastante para conseguir ser entendido(a). |  |  |  |  |  |
| 1. Eu sou dependente de outras pessoas por causa da minha fala. |  |  |  |  |  |
| 1. Eu sinto que as pessoas me consideram menos competente por causa da minha fala. |  |  |  |  |  |
| 1. Eu estou frustrado com minha fala. |  |  |  |  |  |
| 1. Por causa da minha fala, eu evito falar ao telefone. |  |  |  |  |  |
| 1. Por causa da minha fala, descrever acontecimentos é difícil para mim. |  |  |  |  |  |
| 1. Eu me sinto menos competente por causa da minha fala. |  |  |  |  |  |
| 1. Frequentemente me pedem para repetir o que eu disse. |  |  |  |  |  |
| 1. Minha fala afeta minha abilidade em atuar no ambiente de trabalho. * |  |  |  |  |  |
| 1. Por causa da minha fala, participar em conversas rápidas ou complexas é difícil para mim. |  |  |  |  |  |

**Para participantes que não trabalham: A sua fala afeta sua comunicação em ambientes formais / de negócios, como em bancos ou órgãos governamentais ©️ 2016-2026 Vogel, Graf, Synofzik*

**Dysarthria Impact Scale (DIS-6) *Portuguese, “Escala de impacto da disartria”***

| Qual você diria que é a qualidade geral da sua fala? | Muito boa (1) | Boa (2) | Moderada (3) | Ruim (4) | Muito ruim (5) |
| --- | --- | --- | --- | --- | --- |

Por favor, assinale (faça um ‘x’) o quanto você concorda com cada frase abaixo, desde “Concordo totalmente” até “Não concordo nem um pouco”.

|  | Concordo totalmente (1) | Concordo (2) | Nem concordo, nem discordo (3) | Não concordo muito (4) | Não concordo nem um pouco (5) |
| --- | --- | --- | --- | --- | --- |
| 1. Minha fala afeta minha vida social. |  |  |  |  |  |
| 1. Eu me sinto isolado ou solitário por causa da minha fala. |  |  |  |  |  |
| 1. Pessoas que não me conhecem bem tem dificuldade em entender o que eu falo. |  |  |  |  |  |
| 1. Por causa da minha fala, eu evito falar ao telefone. |  |  |  |  |  |
| 1. Por causa da minha fala, descrever acontecimentos é difícil para mim. |  |  |  |  |  |
| 1. Por causa da minha fala, participar em conversas rápidas ou complexas é difícil para mim. |  |  |  |  |  |

*©️ 2016-2026 Vogel, Graf, Synofzik*

**Dysarthria Impact Scale (FULL VERSION) *Polish, “Skala Wpływu Dyzartrii”***

| Jakby Pan/i oceniła ogólnie jakość swojej mowy? | Bardzo dobrze (1) | Dobrze (2) | Średnio (3) | Słabo (4) | Bardzo słabo (5) |
| --- | --- | --- | --- | --- | --- |

Proszę ocenić jak bardzo zgadza się Pan/i z poniższymi stwierdzeniami o Pana/i mowie od “w pełni się zgadzam” do “w ogóle sie nie zgadzam”.

|  | W pełni się zgadzam (1) | Zgadzam się (2) | Ani się zgadzam ani się nie zgadzam (3) | Nie zgadzam się (4) | W ogóle się nie zgadzam (5) |
| --- | --- | --- | --- | --- | --- |
| 1. Z powodu mojej mowy trudno jest mnie zrozumieć w głośnym otoczeniu. |  |  |  |  |  |
| 1. Moja mowa wpływa na moje życie towarzyskie. |  |  |  |  |  |
| 1. Z powodu mojej mowy nie mogę sie komunikować w pełnym zakresie. |  |  |  |  |  |
| 1. Z powodu mojej mowy unikam rozmowy z obcymi. |  |  |  |  |  |
| 1. Czuję się skrępowany/a podczas mówienia. |  |  |  |  |  |
| 1. Czuję, że ludzie traktują mnie inaczej z powodu mojej mowy. |  |  |  |  |  |
| 1. Ludzie nie zaznajomieni ze mną mają trudności ze zrozumieniem mojej mowy. |  |  |  |  |  |
| 1. Wymaga to dużo wysiłku, abym był/a zrozumiany/a. |  |  |  |  |  |
| 1. Jestem zależny od innych z powodu mojej mowy. |  |  |  |  |  |
| 1. Czuję, że ludzie uważają mnie za mniej kompetentnego z powodu mojej mowy. |  |  |  |  |  |
| 1. Jestem zfrustrowany moją mową. |  |  |  |  |  |
| 1. Z powodu mojej mowy unikam rozmów przez telefon. |  |  |  |  |  |
| 1. Z powodu mojej mowy trudno mi jest opisać wydarzenia. |  |  |  |  |  |
| 1. Czuję się mniej kompetentny/a z powodu mojej mowy. |  |  |  |  |  |
| 1. Jestem często proszony/a o powtórzenie tego co powiedziałem/am. |  |  |  |  |  |
| 1. Moja mowa wpływa na moją zdolność do działania w miejscu pracy. * |  |  |  |  |  |
| 1. Z powodu mojej mowy mam trudności w uczestniczeniu w szybkich lub złożonych konwersacjach. |  |  |  |  |  |

**W przypadku osób nie pracujących zawodowo: Czy ich mowa wpływa na komunikację w środowisku urzędowym/ biznesowym? Np. bankowość/załatwianie spraw urzędowych. ©️ 2016-2026 Vogel, Graf, Synofzik*

**Dysarthria Impact Scale (DIS-6) *Polish, “Skala Wpływu Dyzartrii”***

| Jakby Pan/i oceniła ogólnie jakość swojej mowy? | Bardzo dobrze (1) | Dobrze (2) | Średnio (3) | Słabo (4) | Bardzo słabo (5) |
| --- | --- | --- | --- | --- | --- |

Proszę ocenić jak bardzo zgadza się Pan/i z poniższymi stwierdzeniami o Pana/i mowie od “w pełni się zgadzam” do “w ogóle sie nie zgadzam”.

|  | W pełni się zgadzam (1) | Zgadzam się (2) | Ani się zgadzam ani się nie zgadzam (3) | Nie zgadzam się (4) | W ogóle się nie zgadzam (5) |
| --- | --- | --- | --- | --- | --- |
| 1. Moja mowa wpływa na moje życie towarzyskie. |  |  |  |  |  |
| 1. Czuję się odizolowany lub samotny z powodu mojej mowy. |  |  |  |  |  |
| 1. Ludzie nie zaznajomieni ze mną mają trudności ze zrozumieniem mojej mowy. |  |  |  |  |  |
| 1. Z powodu mojej mowy unikam rozmów przez telefon. |  |  |  |  |  |
| 1. Z powodu mojej mowy trudno mi jest opisać wydarzenia. |  |  |  |  |  |
| 1. Z powodu mojej mowy mam trudności w uczestniczeniu w szybkich lub złożonych konwersacjach. |  |  |  |  |  |

*©️ 2016-2026 Vogel, Graf, Synofzik*

**Dysarthria Impact Scale (FULL VERSION) *Czech, “Škála dopadu dysartrie”***

| Jak byste celkově ohodnotil(a) kvalitu své řeči? | Velmi dobrá (1) | Dobrá (2) | Střední (3) | Špatná (4) | Velmi špatná (5) |
| --- | --- | --- | --- | --- | --- |

Ohodnoťte prosím, do jaké míry souhlasíte s následujícími výroky o vaší řeči od „Plně souhlasím“ po „Vůbec nesouhlasím“.

|  | Plně souhlasím (1) | Souhlasím (2) | Ani nesouhlasím, ani souhlasím (3) | Nesouhlasím (4) | Vůbec nesouhlasím (5) |
| --- | --- | --- | --- | --- | --- |
| 1. Kvůli mé řeči je mi v hlučném prostředí obtížně rozumět. |  |  |  |  |  |
| 1. Moje řeč ovlivňuje můj společenský život. |  |  |  |  |  |
| 1. Kvůli své řeči nemohu plně komunikovat. |  |  |  |  |  |
| 1. Kvůli své řeči se vyhýbám rozhovoru s cizími lidmi. |  |  |  |  |  |
| 1. Při mluvení se cítím trapně. |  |  |  |  |  |
| 1. Mám pocit, že se ke mně lidé kvůli mé řeči chovají jinak. |  |  |  |  |  |
| 1. Lidé, kteří mě neznají, mají potíže porozumět mé řeči. |  |  |  |  |  |
| 1. Stojí mě hodně úsilí, aby mi bylo rozumět. |  |  |  |  |  |
| 1. Kvůli své řeči jsem závislý/á na ostatních. |  |  |  |  |  |
| 1. Mám pocit, že mě lidé kvůli mé řeči považují za méně kompetentního/kompetentní. |  |  |  |  |  |
| 1. Jsem frustrovaný/á ze své řeči. |  |  |  |  |  |
| 1. Kvůli své řeči se vyhýbám telefonování. |  |  |  |  |  |
| 1. Kvůli mé řeči je pro mě obtížné popisovat události. |  |  |  |  |  |
| 1. Kvůli své řeči se cítím méně schopný/á. |  |  |  |  |  |
| 1. Často jsem požádán/a, abych zopakoval/a, co jsem řekl/a. |  |  |  |  |  |
| 1. Moje řeč ovlivňuje mou schopnost jednat na pracovišti. * |  |  |  |  |  |
| 1. Kvůli mé řeči je pro mě obtížné účastnit se rychlých nebo složitých rozhovorů. |  |  |  |  |  |

**Pro nepracující účastníky: Má jejich řeč vliv na komunikaci v úředním/obchodním prostředí? Např. v bankovních / úředních záležitostech. ©️ 2016-2026 Vogel, Graf, Synofzik*

**Dysarthria Impact Scale (DIS-6) *Czech, “Škála dopadu dysartrie”***

| Jak byste celkově ohodnotil(a) kvalitu své řeči? | Velmi dobrá (1) | Dobrá (2) | Střední (3) | Špatná (4) | Velmi špatná (5) |
| --- | --- | --- | --- | --- | --- |

Ohodnoťte prosím, do jaké míry souhlasíte s následujícími výroky o vaší řeči od „Plně souhlasím“ po „Vůbec nesouhlasím“.

|  | Plně souhlasím (1) | Souhlasím (2) | Ani nesouhlasím, ani souhlasím (3) | Nesouhlasím (4) | Vůbec nesouhlasím (5) |
| --- | --- | --- | --- | --- | --- |
| 1. Moje řeč ovlivňuje můj společenský život. |  |  |  |  |  |
| 1. Kvůli své řeči se cítím izolovaný/á nebo osamělý/á. |  |  |  |  |  |
| 1. Lidé, kteří mě neznají, mají potíže porozumět mé řeči. |  |  |  |  |  |
| 1. Kvůli své řeči se vyhýbám telefonování. |  |  |  |  |  |
| 1. Kvůli mé řeči je pro mě obtížné popisovat události. |  |  |  |  |  |
| 1. Kvůli mé řeči je pro mě obtížné účastnit se rychlých nebo složitých rozhovorů. |  |  |  |  |  |

*©️ 2016-2026 Vogel, Graf, Synofzik*

**Dysarthria Impact Scale (FULL VERSION) *French, “Échelle d'impact de la dysarthrie”***

| Comment évalueriez-vous la qualité globale de votre parole? | Très bien (1) | Bien (2) | Moyenne (3) | Mauvais (4) | Très mauvais (5) |
| --- | --- | --- | --- | --- | --- |

Veuillez indiquer dans quelle mesure vous êtes d'accord avec les énoncés suivants concernant vos troubles de la parole, en choisissant une réponse allant de « Tout à fait d'accord » à « Pas du tout d'accord ».

|  | Tout à fait d’accord (1) | D’accord (2) | Pas d’avis (3) | Pas d’accord (4) | Pas du tout d’accord (5) |
| --- | --- | --- | --- | --- | --- |
| 1. Mes troubles de la parole m'empêchent de me faire comprendre dans un environnement bruyant. |  |  |  |  |  |
| 1. Ma vie sociale est affectée par mes troubles de la parole. |  |  |  |  |  |
| 1. Mes troubles de la parole m'empêchent de communiquer pleinement. |  |  |  |  |  |
| 1. À cause des troubles de ma parole, j'évite de parler aux personnes que je ne connais pas. |  |  |  |  |  |
| 1. Je me sens embarrassé/e lorsque je parle. |  |  |  |  |  |
| 1. J'ai l'impression que les personnes me traitent différemment du fait de ma parole. |  |  |  |  |  |
| 1. Les personnes qui ne me connaissent pas ont du mal à me comprendre lorsque je parle. |  |  |  |  |  |
| 1. Cela me demande beaucoup d'efforts pour me faire comprendre lorsque je parle. |  |  |  |  |  |
| 1. Je suis dépendant/e des autres à cause de mes troubles de la parole. |  |  |  |  |  |
| 1. J'ai l'impression d'être considéré/e comme moins compétent/e à cause de mes troubles de la parole. |  |  |  |  |  |
| 1. Mes troubles de la parole me frustrent. |  |  |  |  |  |
| 1. À cause de mes troubles de la parole, j'évite de répondre au téléphone. |  |  |  |  |  |
| 1. Mes troubles de la parole m'empêchent de raconter les événements correctement. |  |  |  |  |  |
| 1. Je me sens moins compétent/e à cause de mes troubles de la parole. |  |  |  |  |  |
| 1. On me demande souvent de répéter ce que je dis. |  |  |  |  |  |
| 1. Ma capacité d'accomplir mon travail est affectée par mes troubles de la parole. * |  |  |  |  |  |
| 1. À cause de mes troubles de la parole, j'ai des difficultés à participer aux conversations rapides et complexes. |  |  |  |  |  |

**Pour les participants qui ne travaillent pas : votre parole affecte-t-elle votre communication lors de vos démarches administratives et/ou officielles ? (par ex. : à la banque, dans des administrations, etc.). ©️ 2016-2026 Vogel, Graf, Synofzik*

**Dysarthria Impact Scale (DIS-6) *French, “Échelle d'impact de la dysarthrie”***

| Comment évalueriez-vous la qualité globale de votre parole? | Très bien (1) | Bien (2) | Moyenne (3) | Mauvais (4) | Très mauvais (5) |
| --- | --- | --- | --- | --- | --- |

Veuillez indiquer dans quelle mesure vous êtes d'accord avec les énoncés suivants concernant vos troubles de la parole, en choisissant une réponse allant de « Tout à fait d'accord » à « Pas du tout d'accord ».

|  | Tout à fait d’accord  (1) | D’accord (2) | Pas d’avis (3) | Pas d’accord (4) | Pas du tout d’accord (5) |
| --- | --- | --- | --- | --- | --- |
| 1. Ma vie sociale est affectée par mes troubles de la parole. |  |  |  |  |  |
| 1. Je me sens isolé, ou seul, à cause de mes troubles de la parole. |  |  |  |  |  |
| 1. Les personnes qui ne me connaissent pas ont du mal à me comprendre lorsque je parle. |  |  |  |  |  |
| 1. À cause de mes troubles de la parole, j'évite de répondre au téléphone. |  |  |  |  |  |
| 1. Mes troubles de la parole m'empêchent de raconter les événements correctement. |  |  |  |  |  |
| 1. À cause de mes troubles de la parole, j'ai des difficultés à participer aux conversations rapides et complexes. |  |  |  |  |  |

*©️ 2016-2026 Vogel, Graf, Synofzik*

**Dysarthria Impact Scale (FULL VERSION) *Dutch, “Dysartrie Impact Schaal”***

| Hoe beoordeelt u de kwaliteit van uw spraak in het algemeen? | Heel goed (1) | Goed (2) | Gemiddeld (3) | Slecht (4) | Heel slecht (5) |
| --- | --- | --- | --- | --- | --- |

Geef alstublieft aan in hoeverre u het eens bent met de volgende uitspraken over uw spraakvermogen, van “Helemaal mee eens” tot “Helemaal niet mee eens”.

|  | Helemaal mee eens (1) | Mee eens (2) | Niet mee eens of oneens (3) | Niet mee eens (4) | Helemaal niet mee eens (5) |
| --- | --- | --- | --- | --- | --- |
| 1. Door mijn spraak is het moeilijk me te begrijpen in een lawaaierige omgeving. |  |  |  |  |  |
| 1. Mijn spraak beïnvloedt mijn sociale leven. |  |  |  |  |  |
| 1. Ik kan niet volledig communiceren door mijn spraak. |  |  |  |  |  |
| 1. Ik ontwijk te hoeven spreken met vreemden in verband met mijn spraak. |  |  |  |  |  |
| 1. Ik schaam me om te spreken. |  |  |  |  |  |
| 1. Ik heb het gevoel dat mensen me anders behandelen door mijn spraak. |  |  |  |  |  |
| 1. Mensen die mij niet kennen hebben moeite om mijn spraak te begrijpen. |  |  |  |  |  |
| 1. Het kost veel moeite om mensen me te laten begrijpen. |  |  |  |  |  |
| 1. Ik ben afhankelijk van anderen door mijn spraak. |  |  |  |  |  |
| 1. Ik heb het gevoel dat mensen me minder bekwaam beschouwen door mijn spraak. |  |  |  |  |  |
| 1. Ik ben gefrustreerd door mijn eigen spraak. |  |  |  |  |  |
| 1. Door mijn spraak ontwijk ik telefoneren. |  |  |  |  |  |
| 1. Door mijn spraak is het moeilijk voor mij om gebeurtenissen te beschrijven. |  |  |  |  |  |
| 1. Ik voel me minder bekwaam door mijn spraak. |  |  |  |  |  |
| 1. Er wordt mij vaak gevraagd of ik wil herhalen wat ik zei. |  |  |  |  |  |
| 1. Mijn spraak beïnvloedt mijn capaciteit om te handelen op de werkvloer. * |  |  |  |  |  |
| 1. Door mijn spraak is het moeilijk voor mij om te participeren in snelle of complexe gesprekken. |  |  |  |  |  |

**Voor participanten die niet werken: beïnvloedt hun spraak communicatie in de officiële/zakelijke omgeving? Bv. Bankieren of officiële zaken. ©️ 2016-2026 Vogel, Graf, Synofzik*

**Dysarthria Impact Scale (DIS-6) *Dutch, “Dysartrie Impact Schaal”***

| Hoe beoordeelt u de kwaliteit van uw spraak in het algemeen? | Heel goed (1) | Goed (2) | Gemiddeld (3) | Slecht (4) | Heel slecht (5) |
| --- | --- | --- | --- | --- | --- |

Geef alstublieft aan in hoeverre u het eens bent met de volgende uitspraken over uw spraakvermogen, van “Helemaal mee eens” tot “Helemaal niet mee eens”.

|  | Helemaal mee eens (1) | Mee eens (2) | Niet mee eens of oneens (3) | Niet mee eens (4) | Helemaal niet mee eens (5) |
| --- | --- | --- | --- | --- | --- |
| 1. Mijn spraak beïnvloedt mijn sociale leven. |  |  |  |  |  |
| 1. Ik voel me geïsoleerd of eenzaam door mijn spraak. |  |  |  |  |  |
| 1. Mensen die mij niet kennen hebben moeite om mijn spraak te begrijpen. |  |  |  |  |  |
| 1. Door mijn spraak ontwijk ik telefoneren. |  |  |  |  |  |
| 1. Door mijn spraak is het moeilijk voor mij om gebeurtenissen te beschrijven. |  |  |  |  |  |
| 1. Door mijn spraak is het moeilijk voor mij om te participeren in snelle of complexe gesprekken. |  |  |  |  |  |

*©️ 2016-2026 Vogel, Graf, Synofzik*

**Dysarthria Impact Scale (FULL VERSION) *Turkish, “Dizartri Etki Ölçeği”***

| Konuşmanızın genel kalitesini nasıl değerlendirirsiniz? | Çok iyi (1) | İyi (2) | Orta (3) | Kötü (4) | Çok kötü (5) |
| --- | --- | --- | --- | --- | --- |

Lütfen konuşmanız hakkındaki aşağıdaki ifadelere ne kadar katıldığınızı "Tamamen katılıyorum"dan "Hiç katılmıyorum"a kadar değerlendirin.

|  | Tamamen katılıyorum (1) | Katılıyorum (2) | Ne katılıyorum ne katılmıyorum (3) | Katılmıyorum (4) | Hiç katılmıyorum (5) |
| --- | --- | --- | --- | --- | --- |
| 1. Konuşmam sebebiyle, gürültülü ortamlarda zor anlaşılıyorum. |  |  |  |  |  |
| 1. Konuşmam sosyal hayatımı etkiliyor. |  |  |  |  |  |
| 1. Konuşmam sebebiyle tam olarak iletişim kuramıyorum. |  |  |  |  |  |
| 1. Konuşmam sebebiyle yabancılarla konuşmaktan kaçınırım. |  |  |  |  |  |
| 1. Konuşurken mahcup hissederim. |  |  |  |  |  |
| 1. İnsanlar konuşmam sebebiyle bana farklı davranıyormuş gibi hissederim. |  |  |  |  |  |
| 1. Beni tanımayan / yakınım olmayan insanlar konuşmamı anlamakta zorluk çekerler. |  |  |  |  |  |
| 1. Söylediğimin anlaşılması için çok çaba sarf ediyorum. |  |  |  |  |  |
| 1. Konuşmam sebebiyle başkalarına bağımlıyım. |  |  |  |  |  |
| 1. Konuşmam sebebiyle insanlar benim daha az yetkin olduğumu düşünüyor gibi hissederim. |  |  |  |  |  |
| 1. Konuşmamdan dolayı hayal kırıklığına uğruyorum. |  |  |  |  |  |
| 1. Konuşmam sebebiyle telefonda konuşmaktan kaçınırım. |  |  |  |  |  |
| 1. Konuşmam sebebiyle olayları tarif etmek benim için zor. |  |  |  |  |  |
| 1. Konuşmam sebebiyle daha az yetkin hissediyorum. |  |  |  |  |  |
| 1. Sık sık söylediğimi tekrar etmem istenir. |  |  |  |  |  |
| 1. Konuşmam işyerinde performansımı etkiler.* |  |  |  |  |  |
| 1. Konuşmam sebebiyle hızlı veya karmaşık sohbetlere katılmakta zorluk çekerim. |  |  |  |  |  |

**Çalışmayan katılımcılarda: konuşmaları bürokratik veya iş ortamlarında iletişimlerini etkiliyor mu? Örn, banka, resmi gereklilikler. ©️ 2016-2026 Vogel, Graf, Synofzik*

**Dysarthria Impact Scale (DIS-6) *Turkish, “Dizartri Etki Ölçeği”***

| Konuşmanızın genel kalitesini nasıl değerlendirirsiniz? | Çok iyi (1) | İyi (2) | Orta (3) | Kötü (4) | Çok kötü (5) |
| --- | --- | --- | --- | --- | --- |

Lütfen konuşmanız hakkındaki aşağıdaki ifadelere ne kadar katıldığınızı "Tamamen katılıyorum"dan "Hiç katılmıyorum"a kadar değerlendirin.

|  | Tamamen katılıyorum (1) | Katılıyorum (2) | Ne katılıyorum ne katılmıyorum (3) | Katılmıyorum (4) | Hiç katılmıyorum (5) |
| --- | --- | --- | --- | --- | --- |
| 1. Konuşmam sosyal hayatımı etkiliyor. |  |  |  |  |  |
| 1. Konuşmam sebebiyle izole veya yalnız hissediyorum. |  |  |  |  |  |
| 1. Beni tanımayan / yakınım olmayan insanlar konuşmamı anlamakta zorluk çekerler. |  |  |  |  |  |
| 1. Konuşmam sebebiyle telefonda konuşmaktan kaçınırım. |  |  |  |  |  |
| 1. Konuşmam sebebiyle olayları tarif etmek benim için zor. |  |  |  |  |  |
| 1. Konuşmam sebebiyle hızlı veya karmaşık sohbetlere katılmakta zorluk çekerim. |  |  |  |  |  |

*©️ 2016-2026 Vogel, Graf, Synofzik*

**Supplementary Materials Section 5**

**S5 Scatterplots of associations between DIS-22 and DIS-17 tests**

**
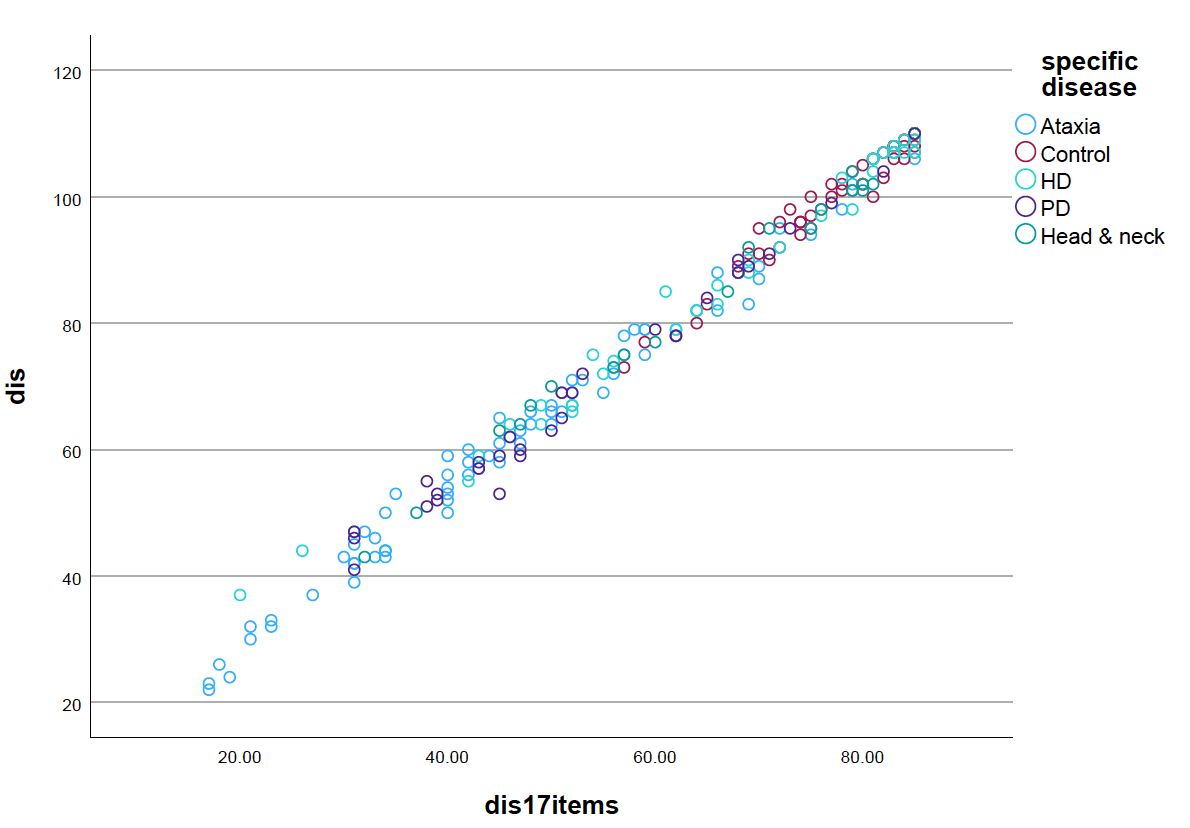
**

DIS-22

**Figure S4.1 Association between DIS-22 and DIS-17.** Note HD= Huntington’s disease, PD=Parkinson’s disease, and ataxia (referring to all dominant and recessive ataxias).

**
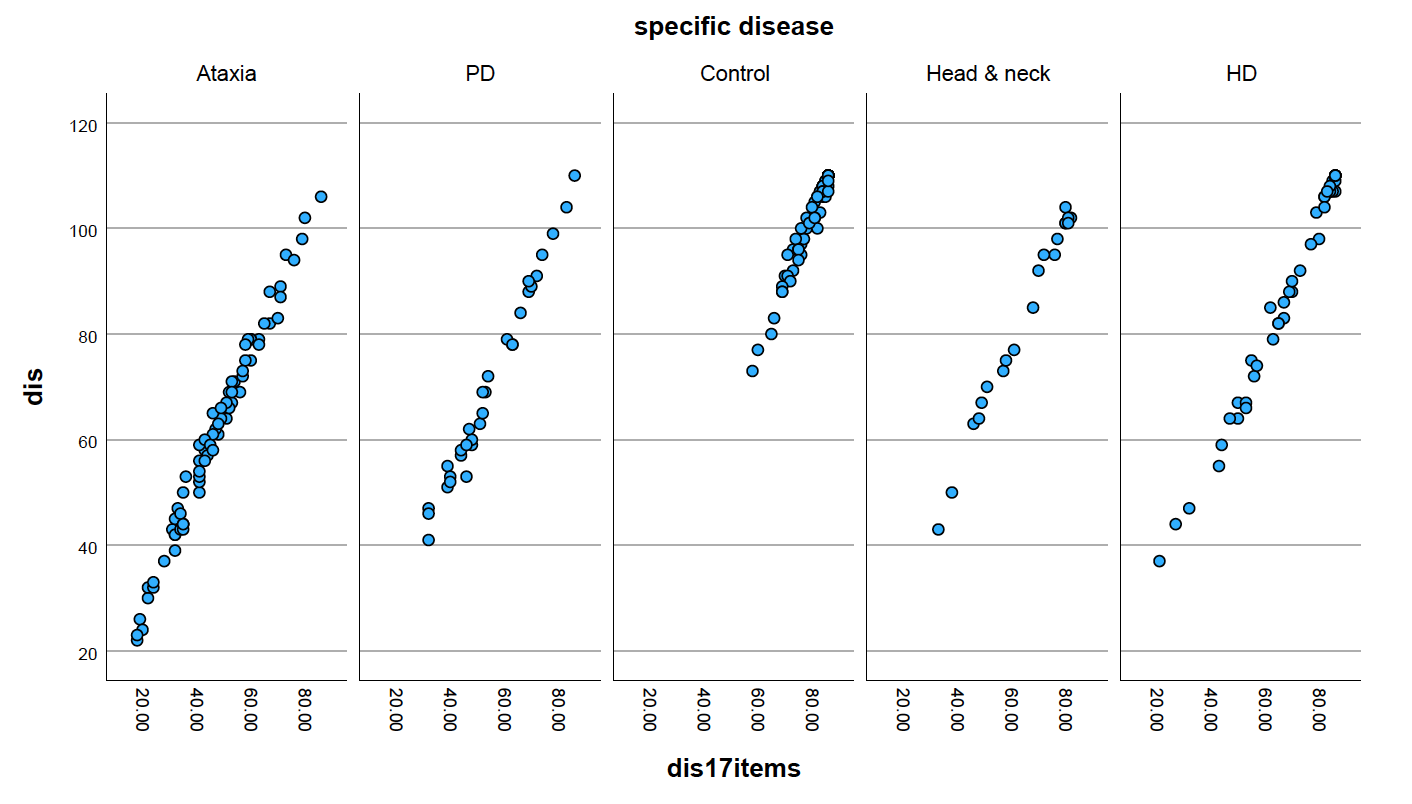
**

DIS-22

**Figure S4.2 Association between DIS-22 and DIS-17 by disease group.** Note HD= Huntington’s disease, PD=Parkinson’s disease, and ataxia (referring to all dominant and recessive ataxias).

**
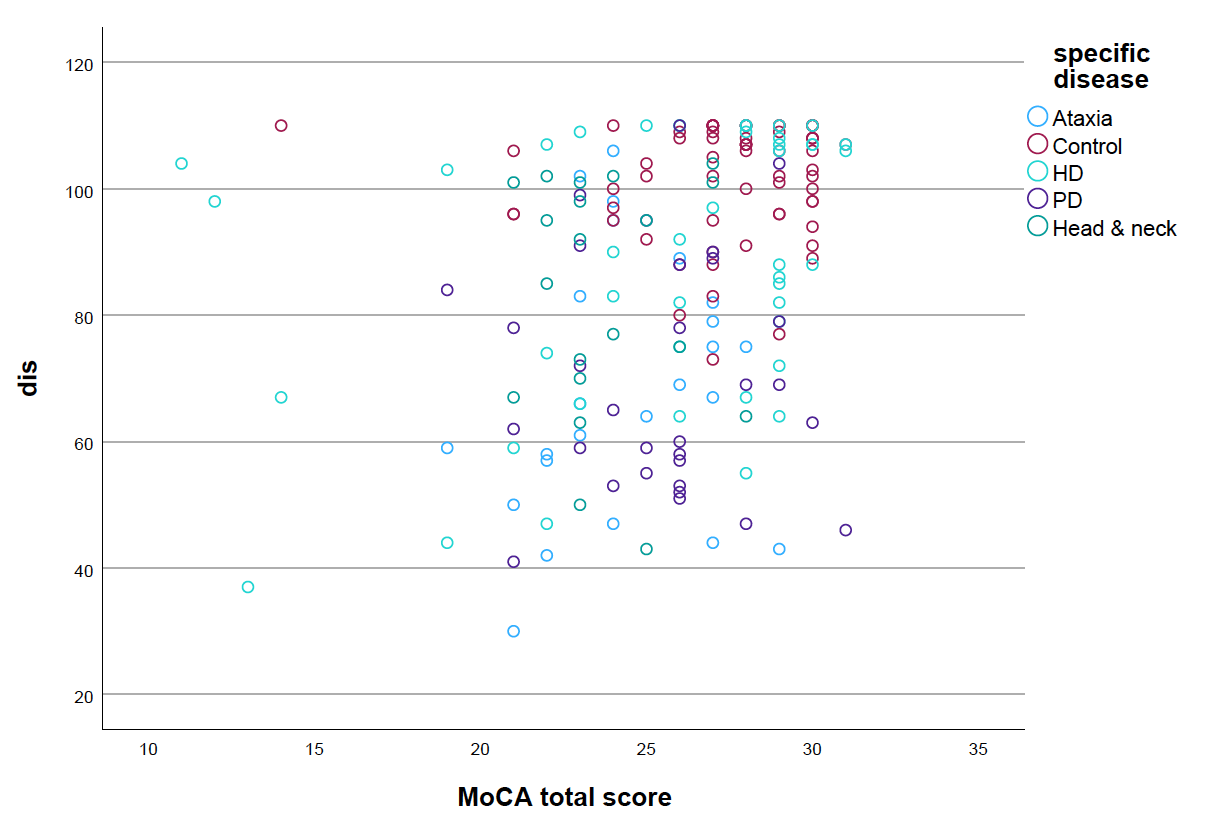
**

DIS-22

**Figure S4.3 Association between DIS-22 and MoCA by disease group.** Note HD= Huntington’s disease, PD=Parkinson’s disease, and ataxia (referring to all dominant and recessive ataxias).

**
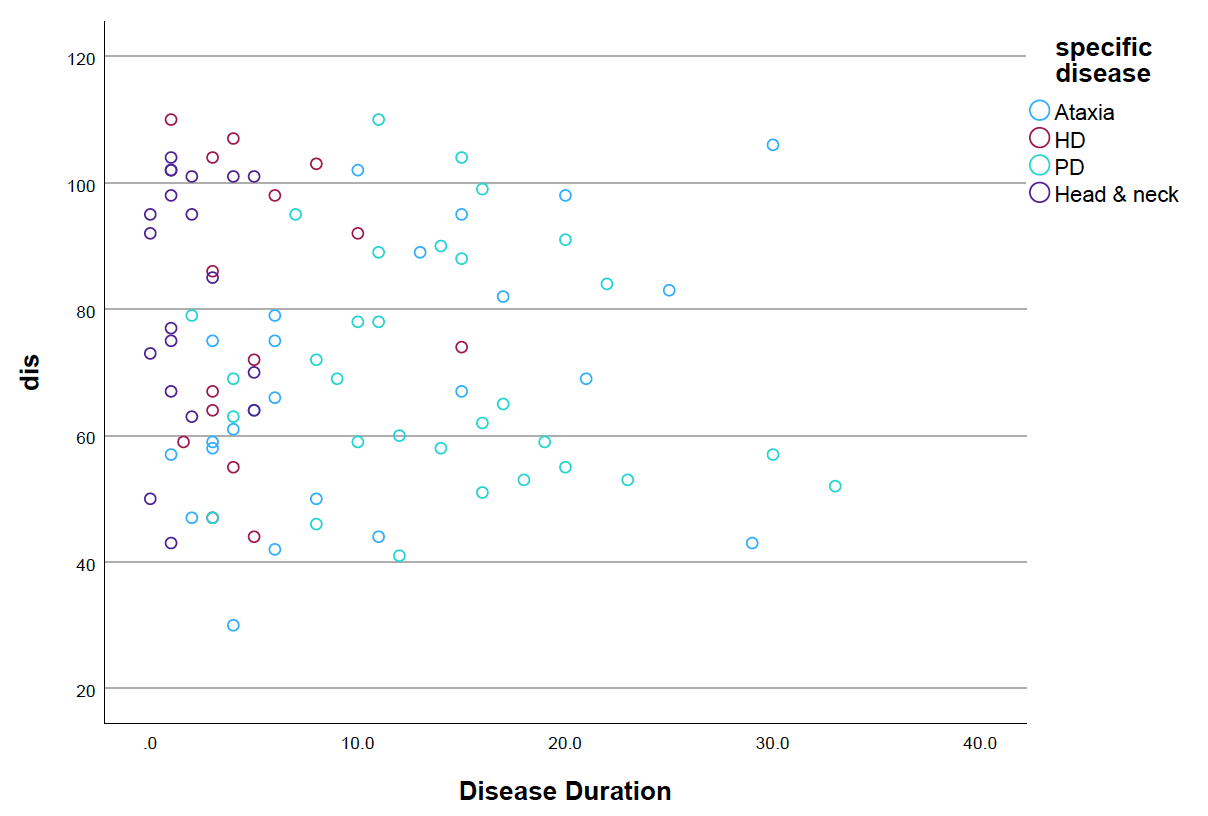
**

DIS-22

**Figure S4.4 Association between DIS-22 and disease duration by disease group.** Note HD= Huntington’s disease, PD=Parkinson’s disease, and ataxia (referring to all dominant and recessive ataxias).

**Table S4.5: Relationship between perceptual dysarthria ratings and DIS scores**

| Perceptual feature and scale (consensus) | DIS-17 | DIS-6 |
| --- | --- | --- |
| Intelligibility rating (Likert) | -0.75 (p<0.001) | -0.75 (p<0.001) |
| Naturalness rating (Likert) | -0.77 (p<0.001) | -0.76 (p<0.001) |
| Intelligibility rating (DME) | 0.78 (p<0.001) | 0.76 (p<0.001) |
| Naturalness rating (DME) | 0.79 (p<0.001) | 0.77 (p<0.001) |

Note: Likert scale ranges from 0-4 with 0 representing unremarkable and 4 representing severe. DME= direct magnitude estimation with 100 representing mild dysarthria and lower values representing more severe dysarthria

**Figure S4.6: Association between DIS-22 and perceptual (A - intelligibility) and (B – naturalness) by disease group.** Note HD= Huntington’s disease, PD=Parkinson’s disease, Ataxia refers to all dominant and recessive ataxias.

**
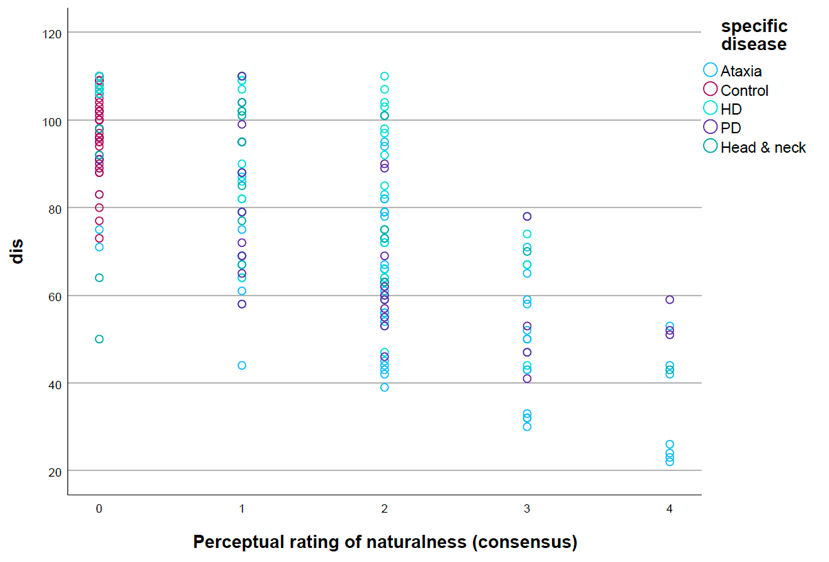

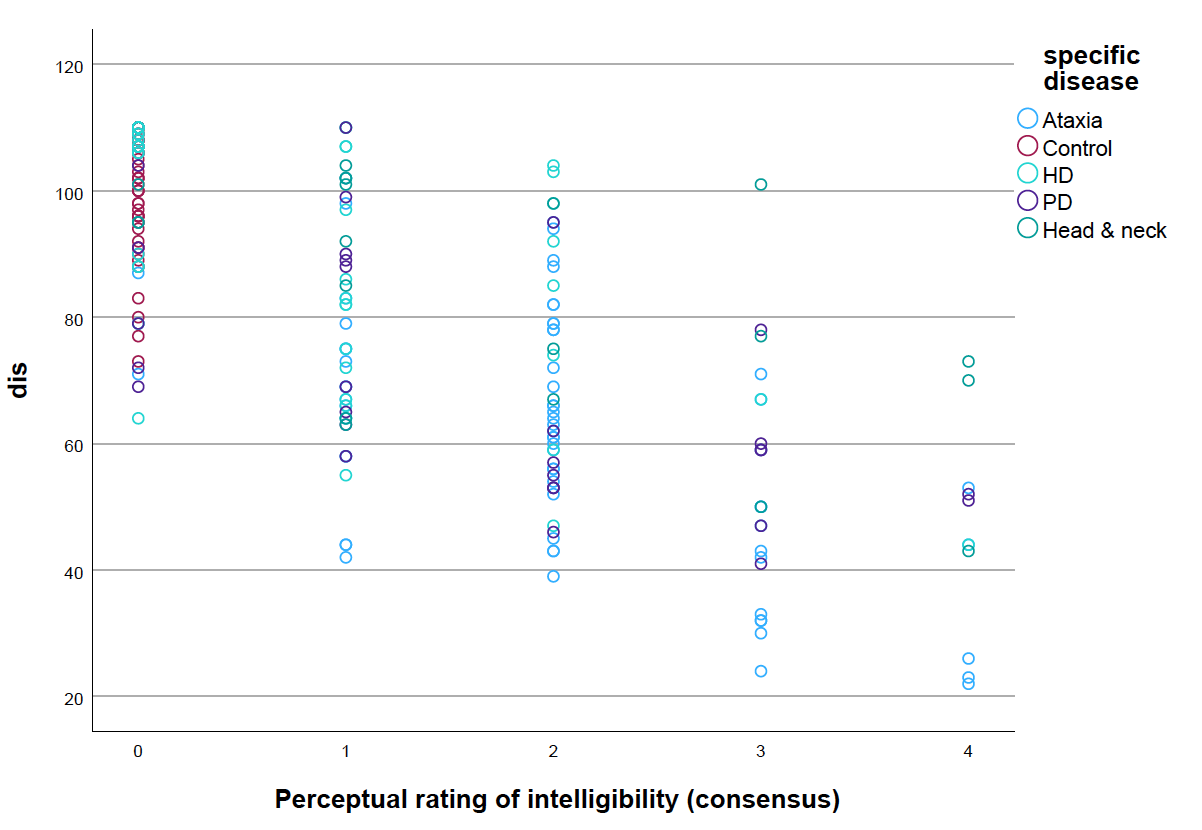

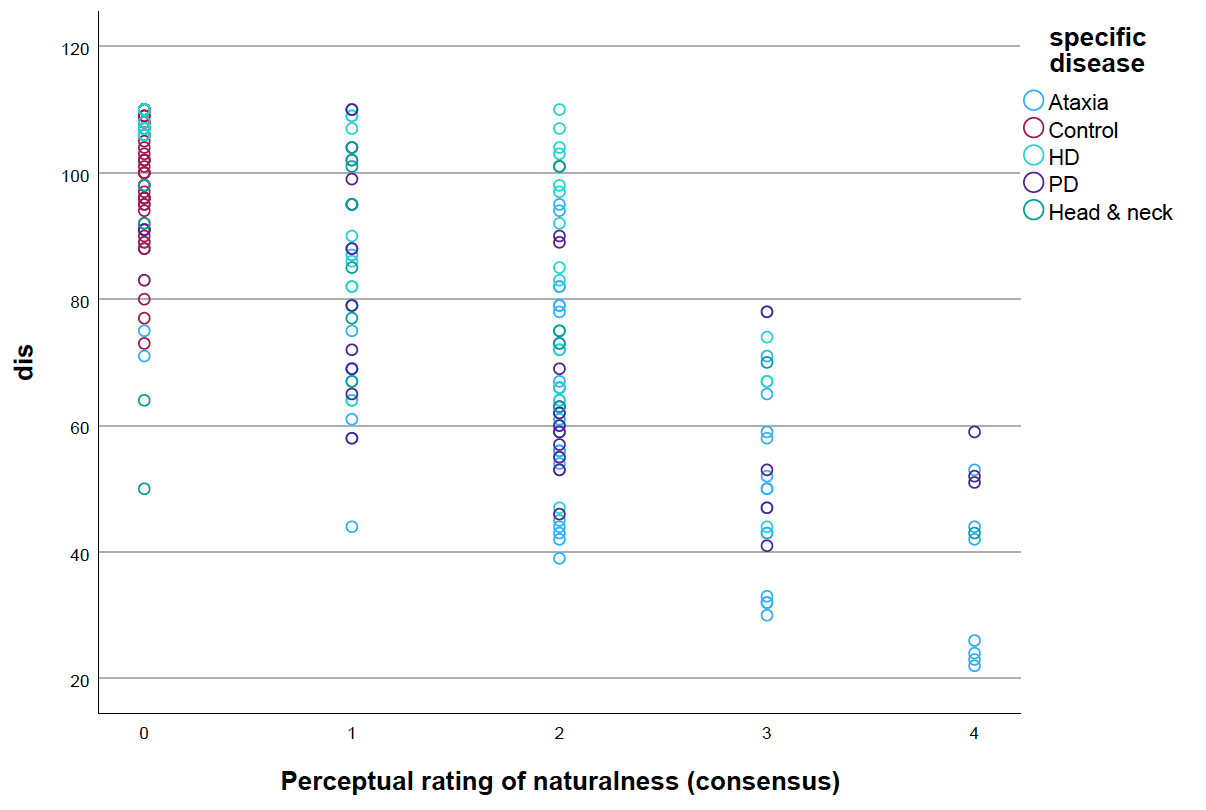
**

A

B

**S5 Minimal detectable change and minimal clinical detectable difference**

**S5.1 Table with WSSD (MDC) and MCID across groups**

| Diagnosis | DIS-6 WSSD | DIS-6 WSSD SE | DIS-6 MCID (0.5√SD) | DIS-6 MCID SE | DIS-17 WSSD | DIS-17 WSSD SE | DIS-17 MCID (0.5√SD) | DIS-17 MCID SE |
| --- | --- | --- | --- | --- | --- | --- | --- | --- |
| Ataxia | 4.29 | 0.5 | 6.07 | 0.36 | 10.63 | 1.24 | 15.04 | 0.88 |
| Huntington's disease | 4.03 | 0.6 | 5.7 | 0.42 | 10.01 | 1.49 | 14.15 | 1.06 |
| Parkinson's disease | 4.02 | 0.72 | 5.68 | 0.51 | 10 | 1.8 | 14.14 | 1.27 |
| Head and neck | 4.02 | 0.9 | 5.68 | 0.64 | 10.02 | 2.24 | 14.18 | 1.58 |
| Healthy controls | 4.05 | 0.49 | 5.73 | 0.34 | 10.16 | 1.22 | 14.37 | 0.86 |

**Note:** Within-subject standard deviation (WSSD) and estimated minimal clinically important difference (MCID) for DIS-6 and DIS-17 across diagnostic groups. SE = standard error, SD = standard deviation**.**

**S5.2 Example Use Case: Evaluating Speech Intervention in Ataxia**

A pharmaceutical company conducts a 12-week trial testing a novel treatment aimed at improving speech function in individuals with spinocerebellar ataxia. Participants complete the DIS-17 at baseline and again at the end of the study. The average DIS-17 score improves from 48.0 to 56.5, a mean change of +8.5 points.

Using the study's data:

- For the ataxia group, the MCID for DIS-17 is ~7.5 points, and the WSSD is ~10.6.
- This 8.5-point improvement exceeds the MCID, suggesting the change is clinically meaningful.
- The change also lies within the expected test–retest variability range (±WSSD), but with statistical confirmation (e.g., paired t-test), it provides evidence that the intervention produced a real, patient-perceived benefit.

If instead the change had been only 4 points:

- It would fall below the MCID, even if statistically significant, and may be deemed clinically negligible.
- Investigators might then conclude the treatment effect is measurable but not yet meaningful to patients.

**Figure S5.3. MCID and WSSD across groups**


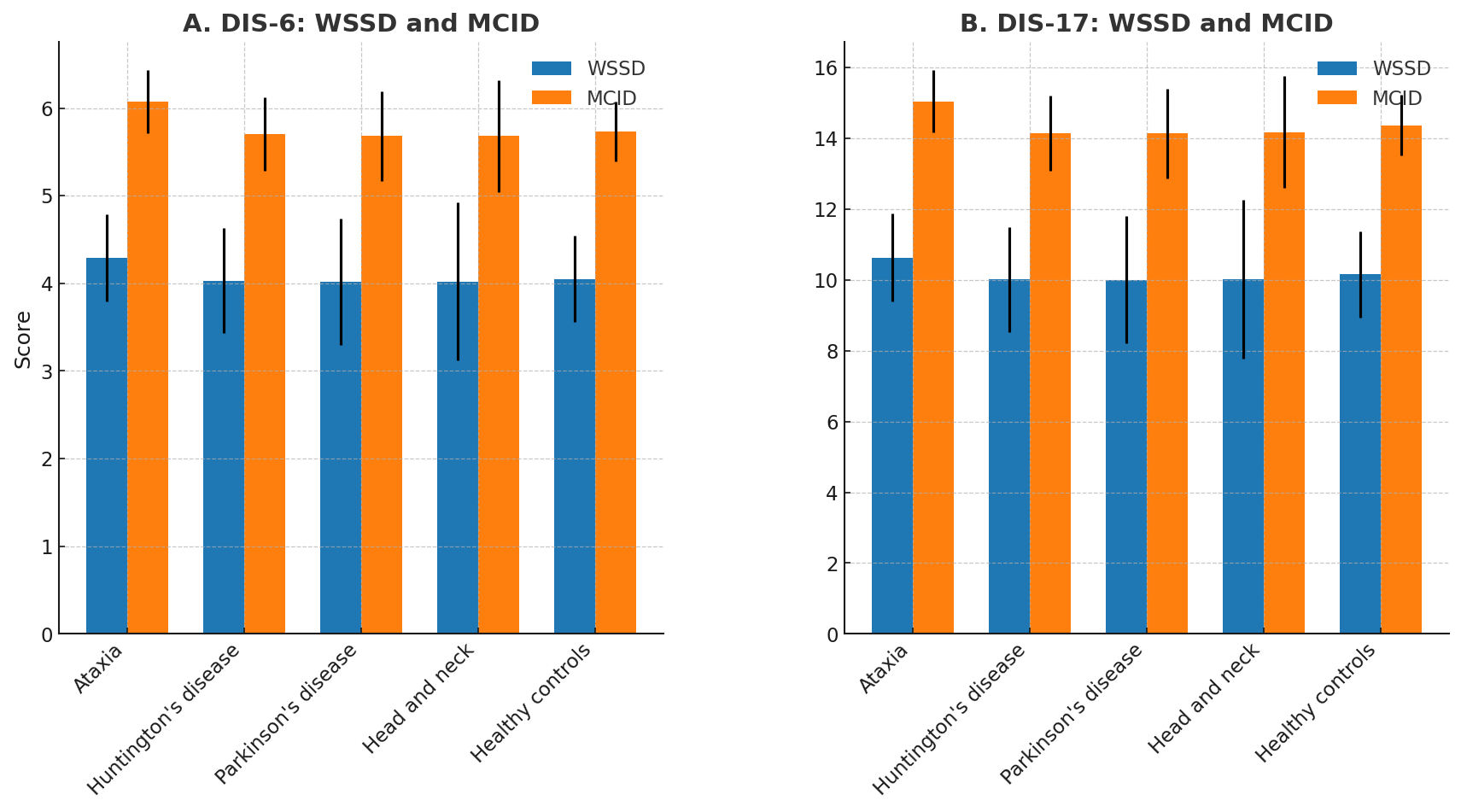


Within-subject standard deviation (WSSD) and estimated minimal clinically important difference (MCID) for DIS-6 and DIS-17 across diagnostic groups.

**S6 Comparing relative difference scores between DIS-6, DIS-17 and VHI across disease groups**

| Disease Group | VHI Mean (95% CI) | DIS-17 Mean (95% CI) | DIS-6 Mean (95% CI) |
| --- | --- | --- | --- |
| Ataxia | 62.8 (57.4–68.1) | 46.1 (42.8–49.4) | 15.8 (14.5–17.1) |
| Control | 19.0 (13.7–24.4) | 79.0 (75.6–82.4) | 28.1 (26.7–29.4) |
| HD | 42.8 (36.2–49.4) | 68.0 (63.8–72.2) | 23.9 (22.2–25.5) |
| PD | 41.8 (33.7–49.9) | 53.6 (48.5–58.7) | 18.1 (16.1–20.1) |
| Head & Neck | 31.4 (21.5–41.3) | 63.4 (57.1–69.7) | 20.5 (18.0–23.0) |

Note: Group comparison (One-way ANOVA) VHI: F(4, 228) = 33.56, p < .001 | DIS-17: F(4, 233) = 51.44, p < .001 | DIS-6: F(4, 233) = 45.95, p < .001. The F tests the effect of specific disease. This test is based on the linearly independent pairwise comparisons among the estimated marginal means.

**Table S7. Internal consistency of Dysarthria Impact Scale versions**

| **Scale version** | **Cohort** | **N** | **Cronbach’s α** |
| --- | --- | --- | --- |
| DIS-22 | Clinical | 169 | 0.970 |
| DIS-17 | Clinical | 169 | 0.968 |
| DIS-6 | Clinical | 174 | 0.917 |
| DIS-22 | Control | 69 | 0.922 |
| DIS-17 | Control | 69 | 0.904 |
| DIS-6 | Control | 70 | 0.812 |

**Supplementary Materials References**

1. Long AF, Hesketh A, Paszek G, Booth M, Bowen A. Development of a reliable self-report outcome measure for pragmatic trials of communication therapy following stroke: the Communication Outcome after Stroke (COAST) scale. Clinical Rehabilitation 2008;22:1083-1094.

2. Baylor C, Yorkston K, Eadie T, Kim J, Chung H, Amtmann D. The Communicative Participation Item Bank (CPIB): item bank calibration and development of a disorder-generic short form. J Speech Lang Hear Res 2013;56:1190-1208.

3. Walshe M, Peach RK, Miller N. Dysarthria Impact Profile: development of a scale to measure psychosocial effects. International Journal of Language & Communication Disorders 2009;44:693-715.

4. Bach KK, Belafsky PC, Wasylik K, Postma GN, Koufman JA. Validity and reliability of the glottal function index. Arch Otolaryngol Head Neck Surg 2005;131:961-964.

5. Yaruss JS, Quesal RW. Overall Assessment of the Speaker's Experience of Stuttering (OASES): Documenting multiple outcomes in stuttering treatment. Journal of Fluency Disorders 2006;31:90-115.

6. Piacentini V, Zuin A, Cattaneo D, Schindler A. Reliability and validity of an instrument to measure quality of life in the dysarthric speaker. Folia Phoniatr Logop 2011;63:289-295.

7. Nanjundeswaran C, Jacobson BH, Gartner-Schmidt J, Verdolini Abbott K. Vocal Fatigue Index (VFI): Development and Validation. Journal of Voice 2015;29:433-440.

8. Jacobson BH, Johnson A, Grywalski C, et al. The Voice Handicap Index (VHI): Development and Validation. American Journal of Speech-Language Pathology 1997;6:66-70.

9. Rosen CA, Lee AS, Osborne J, Zullo T, Murry T. Development and Validation of the Voice Handicap Index-10. The Laryngoscope 2004;114:1549-1556.

10. Gliklich RE, Glovsky RM, Montgomery WW. Validation of a voice outcome survey for unilateral vocal cord paralysis. Otolaryngol Head Neck Surg 1999;120:153-158.

11. Hogikyan ND, Sethuraman G. Validation of an instrument to measure voice-related quality of life (V-RQOL). Journal of Voice 1999;13:557-569.
